# DNA-Binding and Transcription Activation by Unphosphorylated Response Regulator AgrR From *Cupriavidus metallidurans* Involved in Silver Resistance

**DOI:** 10.3389/fmicb.2020.01635

**Published:** 2020-07-17

**Authors:** Md Muntasir Ali, Ann Provoost, Kristel Mijnendonckx, Rob Van Houdt, Daniel Charlier

**Affiliations:** ^1^Research Group of Microbiology, Department of Bioengineering Sciences, Vrije Universiteit Brussel, Brussels, Belgium; ^2^Microbiology Unit, Interdisciplinary Biosciences, Belgian Nuclear Research Centre (SCK CEN), Mol, Belgium

**Keywords:** silver resistance, two-component systems, AgrR, *Cupriavidus metallidurans*, phosphorylation

## Abstract

Even though silver and silver nanoparticles at low concentrations are considered safe for human health, their steadily increasing use and associated release in nature is not without risk since it may result in the selection of silver-resistant microorganisms, thus impeding the utilization of silver as antimicrobial agent. Furthermore, increased resistance to metals may be accompanied by increased antibiotic resistance. Inactivation of the histidine kinase and concomitant upregulation of the cognate response regulator (RR) of the AgrRS two-component system was previously shown to play an important role in the increased silver resistance of laboratory adapted mutants of *Cupriavidus metallidurans*. However, binding of AgrR, a member of the OmpR/PhoP family of RRs with a conserved phosphoreceiver aspartate residue, to potential target promoters has never been demonstrated. Here we identify differentially expressed genes in the silver-resistant mutant NA4S in non-selective conditions by RNA-seq and demonstrate sequence-specific binding of AgrR to six selected promoter regions of upregulated genes and divergent operons. We delimit binding sites by DNase I and in gel copper-phenanthroline footprinting of AgrR-DNA complexes, and establish a high resolution base-specific contact map of AgrR-DNA interactions using premodification binding interference techniques. We identified a 16-bp core AgrR binding site (AgrR box) arranged as an imperfect inverted repeat of 6 bp (ATTACA) separated by 4 bp variable in sequence (6-4-6). AgrR interacts with two major groove segments and the intervening minor groove, all aligned on one face of the helix. Furthermore, an additional in phase imperfect direct repeat of the half-site may be observed slightly up and/or downstream of the inverted repeat at some operators. Mutant studies indicated that both inverted and direct repeats contribute to AgrR binding *in vitro* and AgrR-mediated activation *in vivo*. From the position of the AgrR box it appears that AgrR may act as a Type II activator for most investigated promoters, including positive autoregulation. Furthermore, we show *in vitro* binding and *in vivo* activation with dephosphomimetic AgrR mutant D51A, indicating that unphosphorylated AgrR is the active form of the RR in mutant NA4S.

## Introduction

The numerous applications of silver, a precious metal with recognized antimicrobial and antiviral activity, in industry, commerce, hygiene and healthcare have greatly favored its release and spread in the environment (Silver et al., 2006; Edwards-Jones, 2009; Mijnendonckx et al., 2013a; Huy et al., 2017). Similarly, silver nanoparticles (AgNPs), the most commonly engineered nanomaterial, are incorporated in an ever-growing number of domestic and personal care products (Chaloupka et al., 2010; Duran et al., 2016). Even though silver and AgNPs at low concentrations are considered to pose little risk for human health, their increased release in nature is not without far-reaching consequences. Indeed, increased exposure of microorganisms to enhanced silver concentrations may favor the development of resistance and, in fact, silver-resistant bacteria have already been isolated from environmental, industrial and even clinical environments (Finley et al., 2015). The emergence of such organisms may impede the utilization of silver as antimicrobial agent, especially for medical applications. Furthermore, it has been shown that in some instances, increased metal resistance may be accompanied by an increase in antibiotic resistance (McHugh et al., 1975; Choudhury and Kumar, 1998; Perron et al., 2004; Dieppois et al., 2012; Pal et al., 2017; Wang et al., 2017; Chen et al., 2019).

The cytotoxicity of AgNPs is mainly attributed to the release of Ag^+^ ions, which have many targets and affect many cellular processes, including respiration and membrane function. Intracellular Ag^+^ ions may interact with thiol and other functional groups of various enzymes and proteins, inhibit DNA replication, and promote the generation of reactive oxygen species (ROS) that eventually damage major cellular macromolecules, including nucleic acids, proteins and lipids (Russell and Hugo, 1994; Park et al., 2009; Dakal et al., 2016).

Bacteria generally dispose of a battery of cellular-compartment-specific defense mechanisms to cope with enhanced metal concentrations. These include efflux, sequestration, precipitation, reduction and detoxification (Chandrangsu et al., 2017; Gillet et al., 2019). *Cupriavidus metallidurans*, a Gram-negative β-proteobacterium belonging to the Burkholderiaceae family, is well known for its resistance to multiple metal ions (Mergeay et al., 1985; Janssen et al., 2010; Mergeay and Van Houdt, 2015). The organism possesses, among other mechanisms, several HME-RND type (Heavy Metal Efflux-Resistance, Nodulation and Division) efflux systems and P-type ATPases involved in resistance against several metals including but not limited to copper, zinc, cobalt, cadmium, nickel, silver and gold. Many of these determinants are encoded by megaplasmids in the type strain *C. metallidurans* CH34 (Monchy et al., 2007; Janssen et al., 2010; Bersch et al., 2011; Monsieurs et al., 2011) but are part of the chromid in strain NA4 that is used in this study (Ali et al., 2019). *C. metallidurans* NA4 was isolated from silver-sanitized drinking water obtained from the Russian condensate recycle system aboard the International Space Station (Mijnendonckx et al., 2013b; Monsieurs et al., 2014).

P-type ATPases and HME-RND type efflux systems are generally expressed under the control of one- or two-component systems. Whereas one-component transcription regulators may directly bind a small ligand (inducer or corepressor) that modulates the regulatory output, the prevalent two-component systems (TCSs) make use of signal transduction involving phosphotransfer (Egger et al., 1997). Most simple TCSs consist of just two proteins, a transmembrane histidine kinase (HK) and a cytosolic response regulator (RR) (Salazar and Laub, 2015). In the presence of a specific signal, generally representing a physicochemical stress, the HK catalyzes autophosphorylation by ATP of a conserved histidine residue on the cytosolic part of the membrane protein. This phosphorylated histidine residue is then used as phosphodonor for the *trans*-phosphorylation of a conserved aspartate residue of the RR. The latter are modular proteins generally consisting of a conserved N-terminal receiver domain for interaction with and phosphorylation by the cognate HK, and a variable C-terminal effector or output domain for DNA binding and transcriptional regulation (Gao et al., 2007, 2019). In the absence of the specific signal, a HK may act as phosphatase towards the cognate RR (Kenney, 2010). This must limit inappropriate activation of the RR due to phosphorylation by a non-cognate HK or by small molecules such as acetyl phosphate (McCleary et al., 1993). RRs are generally only active when phosphorylated (Hoch, 2000; Gao et al., 2007), but there are exceptions (see section “Discussion”) and many unphosphorylated RRs do bind DNA, although phosphorylation increases their binding affinity and may in some instances alter binding specificity (Liu and Hulett, 1997; Head et al., 1998; Desai et al., 2016). However, the opposite has been observed as well (Zakikhany et al., 2010).

Recently, we have isolated several laboratory-adapted *C. metallidurans* mutants derived from different strains (CH34, AE104, NA4) that are capable of growing at much higher silver concentrations than the cognate parental strains (Mijnendonckx et al., 2019). Remarkably, none of the well-known silver resistance mechanisms, including the RND efflux pumps encoded by the *silDCBA* and *cusDCBAF* operons, and the P-type ATPase efflux pump encoded by the *cupRAC* operon (Monchy et al., 2007; Bersch et al., 2011; Monsieurs et al., 2011; Mergeay and Van Houdt, 2015), are involved in the adapted response. Common to all investigated mutants is the inactivation of the AgrS HK through single amino acid substitution or transposon insertion. AgrS is part of the two-component regulatory system AgrRS, which was previously uncharacterized and not associated with any phenotype in *C. metallidurans* or other bacteria. AgrS inactivation resulted in enhanced expression of a small set of genes, including *agrR*, suggesting a crucial role for this RR in the enhanced resistance (Mijnendonckx et al., 2019). The importance of AgrR and of PrsQ_1_ and PrsQ_2_, two predicted small periplasmic proteins, in the observed metal-resistant phenotype was further confirmed by the loss of resistance in cognate deletion derivatives of the silver resistant mutant NA4S (Mijnendonckx et al., 2019).

Here, we unravel the molecular basis of AgrR-mediated gene expression culminating in enhanced silver resistance of mutant NA4S. Differentially expressed genes are identified by RNA-seq and AgrR-DNA interactions characterized by use of various *in vitro* techniques, including electrophoretic mobility shift assays (EMSAs), DNase I and chemical “in-gel” footprinting of AgrR-DNA complexes, and premodification binding interference. This allowed the identification of the AgrR binding site in the control region of various differentially expressed target genes, the elaboration of a high-resolution base-specific contact map of the interactions and the deduction of a consensus AgrR box. Furthermore, we assess the importance of phosphorylation/dephosphorylation of AgrR for DNA binding and gene activation with the use of wild-type protein and its phosphomimetic (D51E) and dephosphomimetic (D51A) mutants in *in vitro* DNA binding and *in vivo* reporter gene expression assays, respectively.

## Materials and Methods

### Strains and Growth Conditions

*Cupriavidus metallidurans* was routinely cultured at 30°C in Lysogeny Broth (LB; Thermo Fisher Scientific) or Tris-buffered mineral medium supplemented with 0.2% (wt/vol) gluconate (MM284). *Escherichia coli* was routinely cultured at 37°C in LB. For culturing on agar plates, 2% agar (Oxoid, Belgium) was added. Liquid cultures were grown in the dark on a rotary shaker at 150 rpm. When appropriate, the following chemicals were added to the growth medium at the indicated final concentrations: kanamycin sulfate (50 μg/ml), tetracycline hydrochloride (20 μg/ml) (Tc20), ampicillin sodium salt (100 μg/ml) (PanReac AppliChem, Germany) and chloramphenicol (30 μg/ml), L-arabinose (0.2%) (Sigma-Aldrich, Belgium), and isopropyl β-D-1-thiogalactopyranoside (IPTG; 0.5 mM) (Thermo Fisher Scientific, Belgium), except otherwise stated.

### Plasmid Constructions

All primers used for the amplification of DNA probes and cloning are listed in Supplementary Table 1. All constructs were verified by DNA sequencing. To generate the N-terminal MBP (maltose binding protein)-tagged AgrR overexpression clone pMAL-*agrR*, the *agrR* open reading frame was amplified by PCR using the oligonucleotides agrR_pMAL_FW and agrR_pMAL_RV as primers and *C. metallidurans* NA4 genomic DNA as template. The 0.7 kb amplicon was digested with EcoRI and HindIII, ligated in pMAL-c2X with T4 DNA ligase (Thermo Fischer Scientific, Belgium) and transformed in competent *E. coli* DG1 using ampicillin resistance as selective marker. Correct constructs were transferred to *E. coli* BL21(DE3) for overexpression. To generate the C-terminal His6-tagged AgrR (AgrR-His6) expression clone, the *agrR* coding region was amplified from NA4 genomic DNA with the primer pair agrR_FW/agrR_RV. The amplicon was digested with NdeI and HindIII, and ligated in similarly digested pET24a vector DNA. Correct His6-tagged constructs were transferred to chemically competent *E. coli* SoluBL21 cells (Genlantis, Gene Therapy Systems Inc., United States) for overexpression.

Site-directed mutagenesis to construct the D51A and D51E substitution mutants of AgrR was performed with the Phusion Site-Directed Mutagenesis kit (Thermo Fisher Scientific, Belgium). Expression plasmids (pMAL-*agrR*, pET-*agrR*) were used as template for PCR amplification with the phosphorylated primer pairs agrR_D51A_FW/SDM_RV and agrR_D51E_FW/SDM_RV. The linear amplicons were purified using the Wizard^®^ SV Gel and PCR Clean-up kit (Promega, Netherlands), DpnI treated, self-ligated and transformed into *E. coli* DG1.

Plasmid-based promoter probe reporters P_*prsQ*2_-*gfp*, P_*prsQ*2__(C>*G,G*>*C)*_-*gfp* (substitution of the highly conserved and symmetrically positioned C-G and G-C bps of the inverted repeat to G-C and C-G; transversion mutations), P_*prsQ*2__(C>*T,G*>*A)*_-*gfp* (substitution of the highly conserved and symmetrically positioned C-G and G-C bps of the inverted repeat to T-A and A-T; transition mutations) and P_*prsQ*2__(GT>*CA)*_-*gfp* (a double bp mutation of the consecutive G-C and T-A bps of the promoter proximal direct repeat to C-G and A-T) were constructed by ligating a HindIII/NdeI-digested gBlocks gene fragment into the previously constructed pSCK108-P_*prsQ1*_-*gfp* (Mijnendonckx et al., 2019).

### Overexpression and Purification of AgrR, AgrR_D51A_, and AgrR_D51E_

MBP-tagged proteins were purified by affinity chromatography from 2 L cultures of BL21(DE3) transformants containing plasmid pMAL-*agrR*, pMAL-*agrR*_D51A_, or pMAL-*agrR*_D51E_. Cultures were grown at 37°C in LB medium supplemented with 100 μg/ml ampicillin till OD_600_ of 0.4 –0.6, protein expression was induced with 0.5 mM IPTG (isopropyl-β-D-1-thiogalactopyranoside) and growth continued for 16 h at 20°C. Cells were collected by centrifugation, resuspended in lysis buffer (20 mM Tris–HCl, 200 mM NaCl, 1 mM EDTA, 1 mM DTT, pH 7.4) and disrupted by sonication (20% amplitude, 10 s pulses alternated with 10 s pause) on ice. Cleared cell-free extracts were loaded on an MBPtrap column (GE Lifesciences) and eluted with lysis buffer containing 10 mM maltose. Pooled fractions containing MBP-AgrR proteins were passed through a HiTrap desalting column (GE Lifesciences) to transfer the purified proteins to lysis buffer without maltose.

C-terminal His6-tagged AgrR (AgrR-His6) was purified by a combination of nickel affinity and size exclusion chromatography. Two liters cultures of SoluBL21 transformants containing appropriate pET-*agrR* constructs were grown at 37°C in LB medium supplemented with 50 μg/ml kanamycin. At OD_600_ of 0.6–0.8, 0.3 mM IPTG was added and growth continued overnight at 20°C. Cells were harvested by centrifugation, resuspended in buffer A (50 mM Tris–HCl, 100 mM NaCl, 5% glycerol, pH 7.5) supplemented with 1 mM PMSF (phenylmethylsulfonyl fluoride) and 1 mM benzamidine, and lysed by sonication as indicated above. Cleared cell-free extracts were loaded on a HisTrap HP column (GE Lifesciences) in buffer A supplemented with 30 mM imidazole and eluted with the same buffer containing 500 mM imidazole. Pooled fractions were subsequently loaded on a HiLoad 16/600 Superdex 75 pg (16 mm × 600 mm) column (GE Lifesciences).

The purity of all protein preparations was determined by SDS-PAGE and protein concentrations measured with the Bradford protein assay (BioRad) using BSA (bovine serum albumin) as a standard. All protein concentrations are expressed in monomer equivalents.

### *In vitro* DNA Binding Experiments

All *in vitro* protein-DNA interaction studies were performed with gel-purified (5′-^32^P) single-end labeled probes obtained by PCR amplification with GoTaq ready mix (Promega), genomic DNA of *C. metallidurans* NA4 as template, a pair of suited primers (designated target_probe_FW/RV, see Supplementary Table 1) of which one was 5′-end labeled with (γ-^32^P) ATP (3000 Ci mmol^–1^, Perkin Elmer) and T4 polynucleotide kinase (Fermentas), as described in Nguyen Ple et al. (2010). Binding assays were performed at low concentrations of labeled specific DNA probes (approximately 0.1 nM) and incubated at 22°C in AgrR binding buffer (25 mM Na_2_HPO_4_, 150 mM NaCl, 0.1 mM EDTA, 2 mM MgCl_2_, 1 mM DTT, 10% glycerol, pH 7.0) and in the presence of an excess of non-labeled, non-specific competitor DNA (25 μg/ml sonicated salmon sperm DNA). In EMSAs, free DNA was separated from protein-DNA complexes by gel electrophoresis on 6% polyacrylamide in native conditions. Enzymatic footprinting with DNase I (Roche), chemical “in-gel” footprinting with the copper-phenanthroline ion [(OP)_2_-Cu^+^] (Kuwabara and Sigman, 1987) and premodification binding interference experiments with sparingly modified DNA (Brunelle and Schleif, 1987) were performed as described previously (Wang et al., 1998; Peeters et al., 2004). Sparingly modified DNA for utilization in premodification binding interference assays and reference sequencing ladders was generated as described in (Maxam and Gilbert, 1980).

### RNA-Seq

Gene expression in NA4S, NA4SΔ*agrRS*, and NA4 were compared under non-selective growth conditions. Three independent *C. metallidurans* NA4S, NA4SΔ*agrRS*, and NA4 cultures were allowed to grow until an OD600 of 0.6 was reached. Each culture was subdivided in 2 ml portions and cells were harvested by centrifugation for 2 min at 10,000 *g*. Bacterial pellets were flash frozen by immersion into liquid nitrogen and kept frozen at −80°C at all times. Total RNA was extracted using the Promega SV Total RNA Isolation System kit (Promega, Leiden, Netherlands). RNA sequencing of biological replicates (*n* = 3) (directional mRNA library, RiboZero rRNA depletion and 2× 125 bp paired-end sequencing) was performed by Eurofins genomics (Ebersberg, Germany).

RNA-seq data was processed via the Rsubread-featureCounts-limma/voom pipeline (Rsubread_1.34.7; edgeR_3.26.8; limma_3.40.6) (Law et al., 2016; Liao et al., 2019). Processed data statistics are summarized in Supplementary Table 2. The treat method was used to calculate *p*-values from empirical Bayes moderated *t*-statistics with a minimum log-FC requirement of 1 (McCarthy and Smyth, 2009). A heatmap was constructed using the heatmap.2 program within the gplots package for R.

### *In vivo* Cross-Regulation in *E. coli*

Plasmids P_*prsQ*2_-*gfp*, P_*prsQ*2__(C>*G,G*>*C)*_-*gfp*, P_*prsQ*2__(C>*T,G*>*A)*_-*gfp*, and P_*prsQ*2__(GT>*CA)*_-*gfp* were transformed by electroporation in *E. coli* DG1 carrying pBAD-*agrR*_D51A_*S* (Mijnendonckx et al., 2019). Possible cross-regulation was tested by comparing Gfp production in each construct, either uninduced or induced with 0.2% arabinose. To this end, overnight cultures of three independent colonies [grown in LB in the presence of Km (20 μg ml^–1^) and Cm (30 μg ml^–1^)], were diluted 50 times in the same growth medium with or without 0.2% arabinose. Next, the cultures were incubated for 12 h at 30°C after which the optical density at 600 nm and fluorescence (excitation 480/15; emission 523/20) were measured (CLARIOstar^®^; BMG LABTECH). Data are shown as the ratio of the relative fluorescence to optical density (RFU/OD_600_).

### Other Bioinformatics Tools and Analyses

All TCSs of *C. metallidurans* NA4 were predicted by the web-based server P2RP (Predicted Prokaryotic Regulatory Proteins) (Barakat et al., 2013). Phylogenetic analysis was performed by Neighbor-joining method (Jukes-Cantor model) to create a tree from the predicted OmpR-family protein sequences.

## Results

### Identification of Differentially Expressed Genes in the Silver Resistant Mutant NA4S by RNA-Seq

**FIGURE 1:**
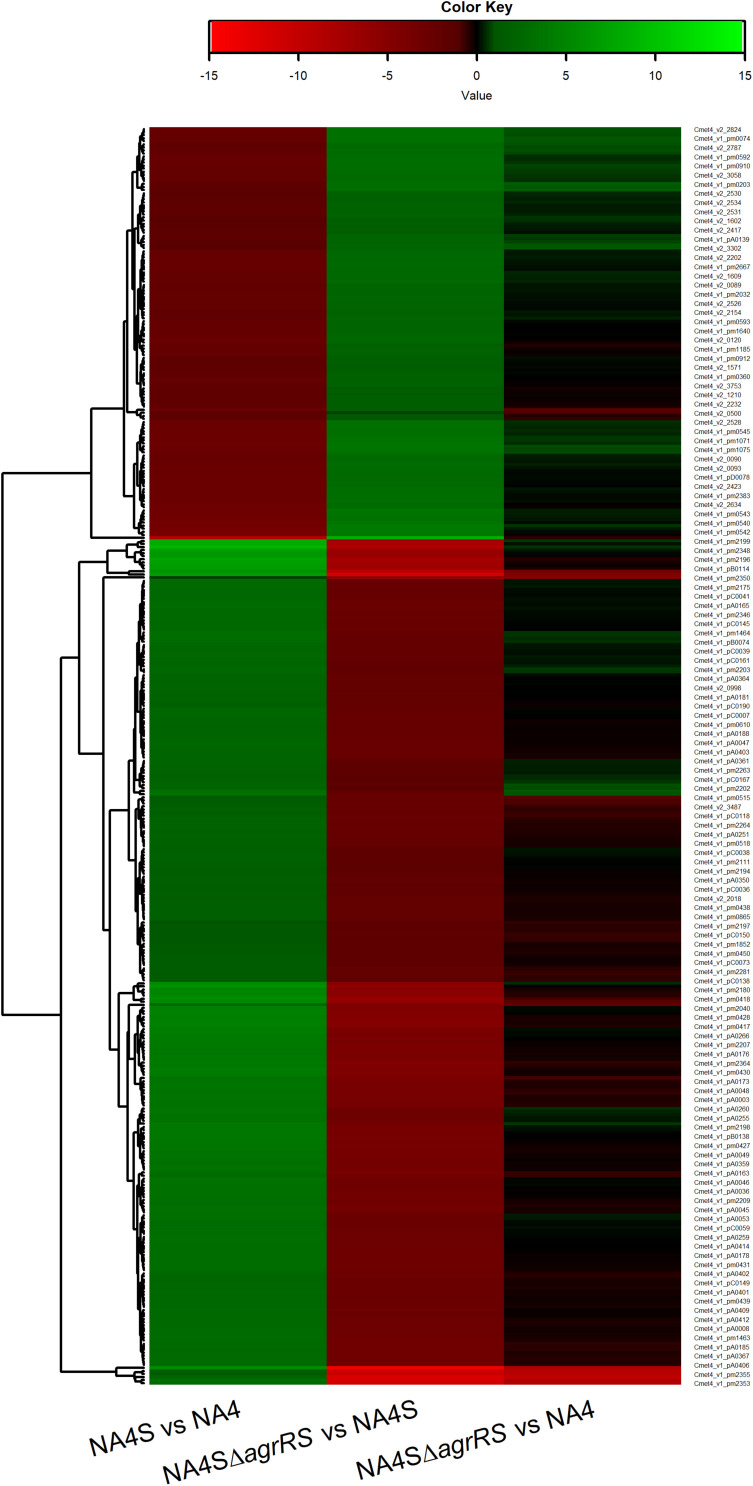
Heatmap of log2-fold changes of differentially expressed genes in *C. metallidurans* NA4S versus NA4, NA4SΔ*agrRS* versus NA4S and NA4SΔ*agrRS* versus NA4 under non-selective growth conditions.

The genome-wide expression analysis of the silver-resistant mutant NA4S was previously performed via the microarray platform of the *C. metallidurans* type strain CH34 (Mijnendonckx et al., 2019). However, taking into account the strain differences (Van Houdt et al., 2018; Ali et al., 2019), we reassessed this more accurately via an RNA-seq approach. As the increased resistance stemmed from natural selection of resistance-conferring mutations (Lenski, 2017), the global shift in transcriptome, resulting from the altered genotype of the evolved strain (NA4S) as compared with the parental strain (NA4), was examined in non-selective conditions (Sandberg et al., 2014; LaCroix et al., 2015; McCloskey et al., 2018). Up- and downregulated genes were selected based on treat method [log2-fold change (≤−1 and ≥1) and significance (*p* < 0.05)], which resulted in 237 up- and 77 down-regulated genes (Supplementary Table 3). From the general overview plot (Supplementary Figure 1), it is noticeable that apparently many genes on the pNA4_A, pNA4_B and pNA4_C plasmids are upregulated. Specifically related to the previously identified targets (Mijnendonckx et al., 2019), *prsQ*_2_ is with a 507-fold increase the most highly upregulated gene in NA4S. The *agrRS* genes (with *agrS* mutated) are also highly upregulated (Table 1). In contrast with the microarray approach, two of the seven genes (v2_1370 and *prsQ*_1_) were not differentially expressed based on RNA-seq. The latter corroborates with previous data from a plasmid-based promoter probe reporter that showed no differential expression in NA4S compared with NA4 (Mijnendonckx et al., 2019). The NA4 systems homologous to known silver detoxification systems (*silDCBA* and *cusDCBAF* operons coding for HME-RND-driven efflux systems, and *cupRAC* coding for a P_IB1_-type ATPase) were not differentially expressed in NA4S (Table 1). The importance of enhanced *agrR* expression in the silver-resistant phenotype was demonstrated by mutant and complementation studies (Mijnendonckx et al., 2019). Therefore, the impact of *agrRS* deletion on the transcriptome was assessed. The latter indicated that deletion of *agrRS* in NA4S affected the transcriptome strongly (Figure 1). Compared with NA4, only 7 genes were downregulated, while it was negatively correlated with that of NA4S (Pearson correlation coefficient of −0.93) (Figure 1).

**TABLE 1:** Log2-fold changes of selected differentially expressed genes in *C. metallidurans* NA4S versus NA4 under non-selective growth conditions.

Locus tag^a^	Product	Log2 FC^b^
v2_1370	Conserved hypothetical, CopQ-like	(−0.55)
v2_0815	Conserved hypothetical, CopQ-like, PrsQ_1_	(0.77)
v1_pm0620	Conserved hypothetical, CopQ-like, PrsQ_2_	8.96
v1_pm0618	Conserved hypothetical, CzcL	2.94
v1_pm0610	Conserved hypothetical, CzcI_2_	2.19
v1_pm2526	Outer membrane protein (porin), OmpC family	5.34
v2_2591	Outer membrane lipoprotein, AgrC	(0.18)
v2_2592	Membrane fusion protein, AgrB	(0.67)
v2_2593	Cation/multidrug efflux pump, AgrA	(0.95)
v2_2595	DNA-binding response regulator, AgrR	5.81
v2_2596	Histidine kinase, AgrS	5.61
v2_0763	Transcriptional regulator, CupR	(−1.26)
v2_0764	P-type ATPase, CupA	(−1.48)
v2_0765	Copper chaperone, CupC	(−0.26)
v1_pm2630	Protein involved in resistance, CusD	(0.15)
v1_pm2629	Outer membrane porin, CusC	ND
v1_pm2628	Membrane fusion protein, CusB	(−0.34)
v1_pm2627	Efflux pump, CusA	(−0.67)
v1_pm2626	Periplasmic copper-binding protein, CusF	ND
v1_pm0440	Transmembrane protein, SilD	(0.16)
v1_pm0441	Outer membrane porin, SilC	(0.23)
v1_pm0442	Membrane fusion protein, SilB	(0.18)
v1_pm0443	Efflux pump, SilA	(0.26)
v1_pm2174	Transmembrane protein, SilD	(−0.08)
v1_pm2172	Outer membrane porin, SilC	(−0.12)
v1_pm2171	Membrane fusion protein, SilB	(−0.44)
v1_pm2170	Efflux pump, SilA	(−0.03)

*^a^NA4 (CmetNA4_) locus tag based on MaGe annotation; ^b^Log2-fold change of gene expression measured by RNA-seq for NA4S compared to its parental strain in non-selective growth conditions (n = 3); Non-significant values (p-value > 0.05) are shown between brackets. ND stands for not determined.*

### AgrR Is a Member of the Large OmpR-Like Family of Response Regulators

To further unravel the role of AgrR, a general overview of the number of TCSs in *C. metallidurans* NA4 was obtained by predicting all RRs and HKs via P2RP (Barakat et al., 2013). A total of 87 RRs and 64 HKs were identified, with the OmpR-family RRs and classic HKs being the most abundant (Supplementary Table 4). Eleven TCSs were identified to be involved in metal resistance (based on homology with known systems of type strain CH34). All except Zni (NarL-family) belong to the OmpR-family. Phylogenetic analysis revealed close homology between the AgrRS, CzcR_1_S_1_, CzcR_2_S_2_, CopR_1_S_1_, and CopR_2_S_2_ systems (Supplementary Figure 2), with the annotation of CzcR_1_S_1_, CzcR_2_S_2_, CopR_1_S_1_, and CopR_2_S_2_ based on their homology to the known pMOL30- and chromid-based Czc system in *C. metallidurans* CH34 (Supplementary Figure 3). Alignment of AgrR with representatives from the OmpR-family identified the conserved phosphorylatable aspartate residue at position 51 (Supplementary Figure 4).

### AgrR Binds Directly to the Control Region of Genes and Operons Differentially Expressed in Mutant NA4S

Previously we have shown that *agrR* is upregulated in NA4S (Table 1) and that heterologous expression of either the mutated *C. metallidurans agrRS*_R414C_ operon (but not parental *agrRS*) or the dephosphomimetic *agrR*_D51A_ allele stimulates expression of a *gfp* reporter transcribed from the *prsQ*_2_ promoter/operator in *E. coli* (Mijnendonckx et al., 2019). These observations strongly suggest that unphosphorylated AgrR is the active DNA-binding form of the RR that stimulates target gene transcription in *C. metallidurans* and that *agrRS* is positively autoregulated. However, a direct interaction of AgrR with either its own control region or control regions of other potential target genes upregulated in NA4S has never been shown.

**FIGURE 2:**
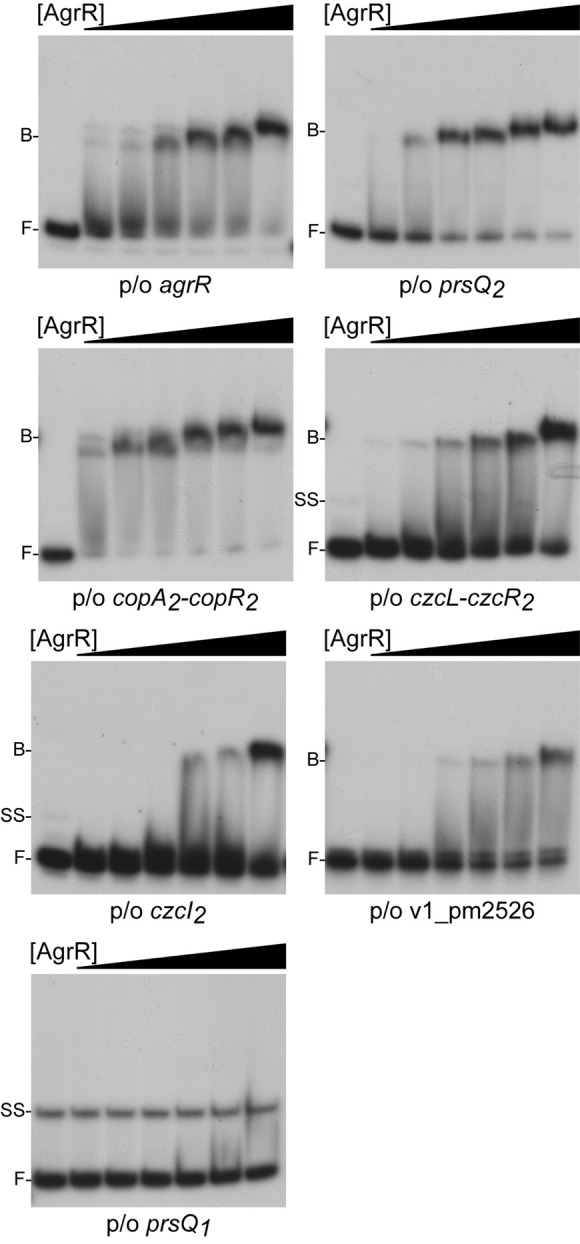
Representative autoradiographs of EMSAs with increasing concentrations of AgrR-His6 (0, 2.5, 5, 10, 15, 20, and 30 μM, expressed in monomer equivalents) binding to approximately 200 bp long control regions of genes and operons upregulated in mutant NA4S in the presence of non-specific competitor DNA (25 μg mL^–1^ sonicated salmon sperm DNA). The position of free DNA (F), single stranded DNA (ss) and protein-DNA complexes (B) is indicated.

Electrophoretic mobility shift assays was used to study *in vitro* binding of purified C-terminal His6-tagged ArgR (AgrR-His6) produced in *E. coli* to approximately 200 bp long 5′-single-end labeled fragments comprising the promoter/operator region of *agrR*, *prsQ*_2_, *czcI*_2_, *ompC*-like (v1_pm2526) and the intergenic *czcL-czcR*_2_ (both divergently transcribed genes are upregulated in NA4S) as well as the *copA*_2_*B*_2_*C*_2_*D*_2_-*copR*_2_ region (of which the *copA*_2_*B*_2_*C*_2_*D*_2_ wing but not *copR*_2_ is upregulated). The *prsQ*_1_ gene (not upregulated but playing an important role in the silver resistant phenotype, see above and Mijnendonckx et al., 2019) was used as negative control. The results revealed binding to all upregulated target genes, whereas at identical protein concentrations no binding was observed to *prsQ*_1_ (Figure 2). The strongest bindings were observed with the *prsQ*_2_ and intergenic *copA*_2_-*copR*_2_ regions (K_*d*_ of 1.6 and 2.1 μM, respectively), followed by *agrR* (16.5 μM) and *czcL-czcR*_2_ (26.5 μM), whereas binding to *czcI*_2_ and *ompC*-like was significantly weaker. Similar DNA binding affinities have been determined by EMSA for a variety of phosphorylated and unphosphorylated RRs (Browning et al., 2004; Ng et al., 2005; Espariz et al., 2007; Nygaard et al., 2010; Giner-Lamia et al., 2012; Mohedano et al., 2016; Hernandez-Eligio et al., 2017).

### Mapping of AgrR Binding Sites by DNase I and Chemical in Gel Footprinting

**FIGURE 3:**
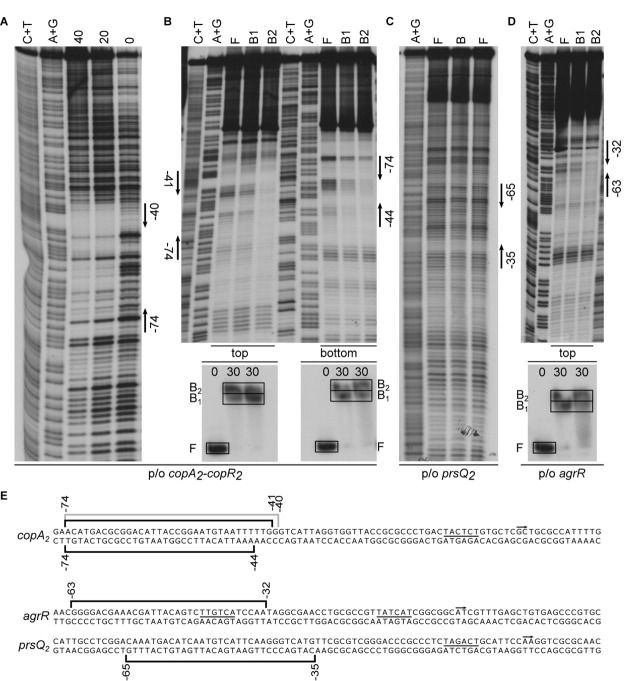
Representative autoradiographs of DNase I and in gel footprinting with the [(OP)_2_-Cu^+^] ion with AgrR-His6 binding to various control regions of genes and operons upregulated in mutant NA4S. **(A)** DNase I footprinting of binding to the intergenic *copA*_2_-*copR*_2_ region (top strand = coding strand with respect to *copA*_2_ revealed). **(B)** In gel footprinting of AgrR-his6 (in μM monomer equivalents, as indicated above the lanes of the EMSA gels) binding to the intergenic *copA*_2_-*copR*_2_ region (both strands revealed) after separation of free (F) and bound (B1, B2) DNA by native acrylamide gel electrophoresis, in gel treatment with the copper-phenanthroline complex, recovery from gel and separation of the reaction products by gel electrophoresis in denaturing conditions. **(C)** In gel footprinting of AgrR-his6 (in μM monomer equivalents, as indicated above the lanes of the EMSA gels) binding to the *prsQ*_2_ operator (bottom strand = non-coding revealed) and **(D)** with the own *agrR* control region (top = coding strand revealed). Arrows delimit the regions of protection. A+G and C+T represent the Maxam–Gilbert sequencing ladders. **(E)** Sequences of the control regions with indication of the regions of protection against enzymatic (gray line) and/or chemical cleavage (black line). The start of transcription (+1, short arrow) and presumed –10 promoter element (underlined) are indicated.

DNase I footprints of AgrR-His6 binding to the intergenic *copA*_2_-*copR*_2_ region revealed a 35-nt long region of protection on the top strand (coding for *copA*_2_), extending from position −74 to −40 with respect to the *copA*_2_ start of transcription (−60 to −26 with respect to the *copR*_2_ initiation site) (Figures 3A,E). In gel footprinting of protein-DNA complexes formed with the same intergenic region using the copper-phenanthroline ion [(OP)_2_-Cu^+^] revealed on both strands a 31–34-nt region of protection that fully coincides with the DNase I footprint (Figures 3B,E). Similarly, in gel footprinting with AgrR-His6 revealed protection of a 31-nt long stretch extending from position −65 to −35 on the bottom (non-coding) strand of the *prsQ*_*2*_ operator (Figures 3C,E), and of 32-nt of the own control region (from −63 to −32 on the coding strand) (Figures 3D,E).

### High Resolution Contact Probing of Dephosphomimetic MBP-AgrR_D51A_ Binding to the Own Control Region

**FIGURE 4:**
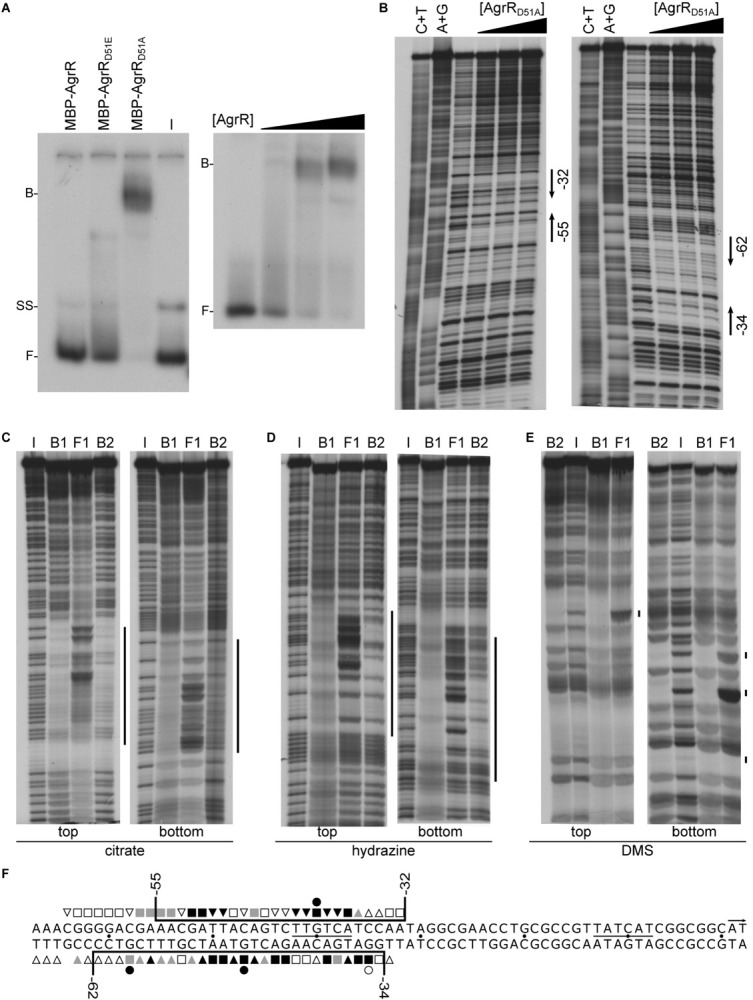
Representative autoradiographs of *in vitro* binding assays with MBP-tagged AgrR proteins binding to the own control region. **(A)** EMSAs with identical amounts (5.85 μM) of MBP-AgrR, MBP-AgrR_D51E_, and MBP-AgrR_D51A_ binding (left hand panel) and increasing concentrations (ranging from 0.3 to 5.85 μM, expressed in monomer equivalents) of MBP-AgrR_D51A_ (right hand panel). **(B)** DNase I footprinting with both top and bottom strand revealed. Protein concentrations used are 0, 2.9, 4.4, and 5.9 μM (expressed in monomer equivalents). Regions of protection are delimited with black colored arrows. A+G and C+T are the Maxam–Gilbert sequencing ladders. **(C–E)** Premodification binding interference experiments with sparingly modified *agrR* operator DNA (top and bottom strand revealed) after treatment with citrate (depurination), hydrazine (depyrimidination) and dimethylsulfate (purine methylation), respectively. Modified operator DNA was incubated with various concentrations of MBP-AgrR_D51A_ resulting in about 50% (B1) and 80% (B2) bound DNA (migrating with the same velocity), bound DNA was separated from free DNA by native gel electrophoresis, the different populations extracted from gel and after cleavage at modified positions with piperidine, equal amounts of the reaction products were separated by gel electrophoresis in denaturing conditions. I, input DNA (no protein added); F, free DNA; B1 and B2, bound DNA. Regions in which base removal or modification interferes with complex formation are indicated with a black colored line. **(F)** Nucleotide sequence of the *agrR* control region with indication of the region protected against DNase I cleavage (black colored line) and positions that upon base removal or guanine premethylation interfere strongly (black-filled symbols), moderately (gray-filled symbols) or weakly (black open symbols) with complex formation. Numbers indicate positions with respect to the start of transcription (+1).

To evaluate the importance of aspartate D51 on the DNA-binding capacity of AgrR, we tested *in vitro* binding of N-terminal MBP-tagged AgrR (MBP-AgrR) and its phosphomimetic (D51E) and dephosphomimetic (D51A) derivatives to the own control region. MBP-AgrR was used because overproduction and purification of the His6-tagged AgrR mutant proteins resulted in very low yields and poorly soluble protein preparations. In EMSA, the strongest binding (K_*d*_ 1.6 μM) was observed with AgrR_D51A_ (Figure 4A), whereas less binding was observed with wild-type AgrR and the phosphomimetic derivative AgrR_D51E_, indicating that unphosphorylated AgrR is the most active DNA-binding form of AgrR, an observation that is in agreement with the *in vivo* reporter gene assays (Mijnendonckx et al., 2019).

DNase I footprinting of MBP-AgrR_D51A_ binding to the own control region revealed the protection on both strands of a 24–30-nt long stretch extending from position −55 to −32 with respect to the transcription initiation site on the top (coding) strand and from position −62 to −34 on the bottom (template) strand (Figures 4B,E). It is worth noticing that no hyperreactive cleavage sites, an indication of strong DNA deformation upon protein binding, were observed. Identical footprints were generated with MBP-AgrR_D51A_ and AgrR-His6 binding to the own control region and to the intergenic *copA*_2_-*copR*_2_ region (Supplementary Figure 5) and no binding or protection was observed with MBP alone. Both observations indicate that in spite of its large size, the MBP-tag does not affect the results.

Missing contact probing (Brunelle and Schleif, 1987) was used to identify purine and pyrimidine residues on both strands of the operator that significantly contribute to the energy of protein-DNA complex formation (Figure 4). Therefore, sparingly (statistically one base per DNA molecule) depurinated (citrate treated) or depyrimidated (hydrazine treated) single 5′-end labeled DNA was incubated with different concentrations of MBP-AgrR_D51A_, bound DNA (B1, B2) separated from free DNA (F) by EMSA, the various forms extracted from gel, the backbone cleaved at modified positions with piperidine at high temperature (90°C) and the reaction products analyzed by gel electrophoresis in denaturing conditions. In this approach, DNA molecules missing a base that significantly contributes to the energy of complex formation are expected to be underrepresented in the bound form(s) and overrepresented in the free form, whereas molecules missing a base that is not contacted by AgrR are expected to be evenly represented in the bound and free forms. The results (Figures 4C,D) indicate that all strong and moderate negative interference signals can be attributed to purine and pyrimidine residues located within the region of protection as determined by footprinting, but they do not all contribute to the same extent (Figure 4F). Furthermore, additional weak interference signals were observed on both strands in an approximately 6–9-nt long promoter distal but immediately flanking region (from −59 to −67) (Figure 4F).

Premethylation binding interference was used to gather further information on groove-specificity of AgrR binding. DMS (dimethylsulfate) was used to sparingly methylate guanine residues at the N7 position, protruding in the major groove, and adenine residues at the N3 position, protruding in the minor groove (Siebenlist and Gilbert, 1980). Subsequent cleavage of the backbone at methylated purines by piperidine after separation of bound and free DNA forms by EMSA and extraction from gel essentially reveals methylated guanines, whereas the reaction rate of adenines is low in these conditions. Methylation adds a relatively large methyl group and a positive charge to the purine base, and eliminates a potential acceptor group for hydrogen bonding. As a consequence, negative interference signals of methylated guanine residues may be attributed to local steric exclusion from binding or to changes in the resonance state of the purine ring such that the protein does no longer recognize the methylated residue. The results (Figures 4E,F) revealed strong negative interference signals for one guanine residue (G_–40_) of the top strand and two guanine residues (G_–47_, G_–58_) separated by one full helical turn of the bottom strand. An additional weaker interference signal was observed at G_–35_ of the bottom strand. From the position of all negative interference signals we may conclude that AgrR interacts with three successive major groove segments of the operator, all aligned on the same face of the DNA helix. As no interference was observed upon methylation of guanine residues located in major groove segments situated on the opposite face of the helix, we may conclude that AgrR interacts mainly with one face of the DNA, a characteristic of proteins bearing a HTH (helix-turn-helix) motif for DNA binding (Aravind et al., 2005).

### Alignment of AgrR Binding Sites and Deduction of the AgrR Consensus Binding Sequence and Sequence Logo

**FIGURE 5:**
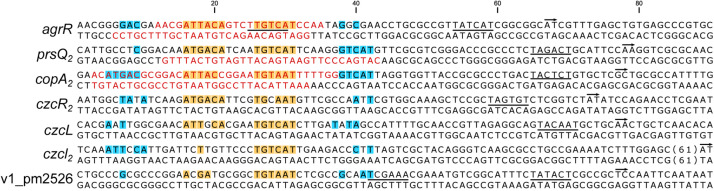
Nucleotide sequences of promoter/operator regions of genes and operons identified as AgrR targets by EMSA (Figure 2). Sequences were aligned on basis of experimentally identified regions of AgrR binding (red colored letters) for *agrR*, *prsQ*_2_ and the intergenic *copA*_2_-*copR*_2_ region (Figures 3, 4), and on basis of sequence conservation with these regions for the other targets. The start of transcription (+1) is indicated with a short arrow, the presumed –10 promoter element is underlined. Highly conserved sequences are highlighted in yellow for the core binding site comprising an inverted repeat and in blue for potential additional direct repeats of the half site.

**FIGURE 6:**
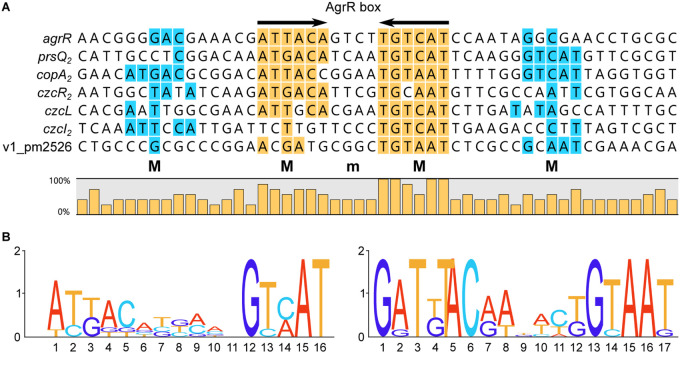
**(A)** Sequence alignment of AgrR binding sites with indication of frequencies of occurrence of the different bases and deduced 16-bp consensus ArgR binding site (AgrR box) consisting of 6-bp inverted repeats separated by 4 bp (6-4-6). Palindromic sequences are highlighted in yellow, direct repeats of the half site in blue. M indicates binding to major groove segments aligned on the same face of the helix, m represents the intervening minor groove segment of the core binding site, aligned on the same face. **(B)** Sequence logo of the core ArgR binding site as deduced from the experimental results (left part) and sequence logo as established from *in silico* predicted ArgR binding sites (Mijnendonckx et al., 2019). Logos from multiple sequence alignments were generated using WebLogo (Crooks et al., 2004).

Figure 5 presents an alignment of operator sequences to which AgrR binds *in vitro* as demonstrated by EMSA (Figure 2) with experimentally delimited regions of protection by footprinting in the *agrR*, *prsQ*_2_, and *copA*_2_-*copR*_2_ control regions and shown to be important for complex formation with the *agrR* binding site by premodification binding interference (Figures 3, 4F). From the alignment and the degree of sequence conservation (Figures 5, 6), it appears that naturally occurring ArgR binding sites comprise an imperfect inverted repeat of 16 bp composed of 6-bp repeats separated by a 4-bp stretch variable in sequence, with as consensus ATKACANNNNTGTMAT. This consensus sequence matches the previously *in silico* established AgrR binding site (Figure 6; Mijnendonckx et al., 2019) but exhibit a higher degree of sequence conservation in the promoter proximal half-site of the inverted repeat than in the promoter distal part. From the premethylation binding interference experiments (Figures 4E,F) it may be concluded that of this sequence the two conserved outer major groove segments aligned on one face of the helix are contacted by AgrR. Furthermore, an additional imperfect direct repeat of a half-site may be observed slightly up and/or downstream of the inverted repeat, especially in the higher affinity targets *copA*_2_, *prsQ*_2_, and *agrR*. It is worth noticing that both base removal (citrate, hydrazine) and premethylation (DMS) of the promoter distal direct repeat in the *agrR* operator negatively affects complex formation (Figure 4F).

**FIGURE 7:**
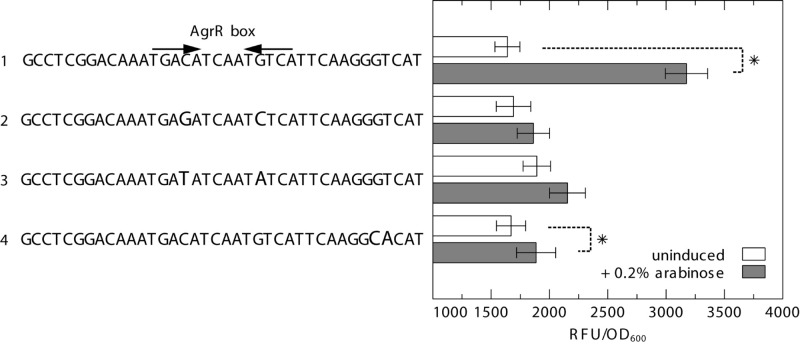
GFP fluorescence in *E. coli* expressing GFP from the *prsQ*_2_ promoter with (1) the native AgrR box, (2) C>G and G>C substitution of the highly conserved and symmetrically positioned C-G and G-C bps of the inverted repeat to G-C and C-G (transversion mutations), (3) C>T and G>A substitution of the highly conserved and symmetrically positioned C-G and G-C bps of the inverted repeat to T-A and A-T (transition mutations), and (4) GT>CA substitution of the consecutive G-C and T-A bps of the promoter proximal direct repeat to C-G and A-T, in the absence or presence (induced with 0.2% arabinose) of AgrR with a dephosphomimetic mutation (pBAD-*agrR*_D51A_*S*). The reporter signal (RFU) was normalized to the cell density (OD600). The average values of three independent experiments with standard deviations are shown. Asterisk indicates a significant difference between the induced and uninduced control, determined by a *t*-test with a *p* < 0.05.

To further analyze the importance of both inverted- and direct repeats in AgrR-mediated gene activation, we used the previously described heterologous dual expression/reporter system (Mijnendonckx et al., 2019) in which a *prsQ*_2_ promoter-*gfp* fusion (P_*prsQ*2_-*gfp*) is combined with an arabinose inducible *agrR*_D51A_ mutant expression vector. The results (Figure 7) clearly demonstrate that substitution of the highly conserved and symmetrically positioned C-G and G-C bps of the inverted repeat to G-C and C-G, respectively (transversion mutations), or T-A and A-T (transition mutations) nearly completely abolish the arabinose induced activation observed with the wild-type *prsQ*2 promoter/operator. Similarly, a double bp mutation of the consecutive G-C and T-A bps of the promoter proximal direct repeat to C-G and A-T, respectively, largely affected the AgrR_D51A_-mediated activation. Therefore, it may be concluded that both inverted- and direct repeats contribute to AgrR-mediated gene activation.

## Discussion

We show that RR AgrR of *C. metallidurans* directly binds to the own control region and the promoter/operator of various genes and operons that are upregulated in silver-resistant mutant NA4S, which carries a mutated cognate HK AgrS. AgrR is a member of the large family of OmpR/PhoP-like RRs, which are generally only active in the phosphorylated form and dephosphomimetic mutations of the conserved aspartate residue within the receiver domain are generally detrimental to function (Martinez-Hackert and Stock, 1997; Head et al., 1998; Gardino and Kern, 2007). However, the opposite was observed for AgrR. In most members of both the PhoP/OmpR and Fix/NarL classes of RRs, interaction between the receiver and output domain results in inhibition of dimerization and DNA binding, which may be reversed by phosphorylation or removal of the N-terminal receiver domain (Martinez-Hackert and Stock, 1997; Maris et al., 2002; Feng et al., 2004; Nowak et al., 2006; Friedland et al., 2007; Giner-Lamia et al., 2012). However, there are exceptions (Desai and Kenney, 2017) as further developed below and demonstrated for instance for CsgD, HoxA, PilR, AlgB, AlgR and DegU. CsgD controls biofilm formation in *Salmonella enterica* subsp. *enterica* serovar Typhimurium and is a member of the FixJ/NarL family of RRs, which is the second most abundant family of RRs containing a winged HTH motif (Gao et al., 2007). Unphosphorylated CsgD was shown to bind specifically to the *csgBA* (curli fiber synthesis) and *adrA* (diguanylate cyclase) promoter regions and to stimulate transcription *in vitro*, whereas *in vitro* phosphorylation of CsgD with acetylphosphate decreased its DNA binding affinity (Zakikhany et al., 2010). Furthermore, both the dephosphomimetic (D59N) and phosphomimetic (D59E) *csgD* mutants were shown to be strongly affected in the gene activating capacity due to reduced protein stability and DNA binding in case of D59E (Zakikhany et al., 2010). A deficiency of phosphorylation of HoxA, involved in H_2_ sensing in *Alcaligenes eutrophus* (later renamed *Cupriavidus necator*), by either a mutation in the cognate HK HoxJ or substitution of the conserved phosphorylatable aspartate residue of HoxA, was shown to result in increased target site activation (Lenz and Friedrich, 1998). Similarly, the dephosphomimetic mutant (D53N) of PilR from *Geobacter sulfurreducens* was shown to be more potent in activating *pilA* expression than wild-type PilR, strongly suggesting that the unphosphorylated RR is the active form involved in synthesis of the pilus subunit (Hernandez-Eligio et al., 2017). Nonphosphorylatable AlgB and AlgR were shown to be still active in promoting alginate biosynthesis in mucoid *Pseudomonas aeruginosa* (Ma et al., 1998) and DegU of *Bacillus subtilis* was shown to be active in both the phosphorylated and unphosphorylated form. Unphosphorylated DegU activates late competence genes, whereas phosphorylated DegU acts as a rheostat that senses and responds to changes in the environment and enables the integration of various cellular responses, including genetic competence, swarming motility, biofilm formation and exoprotease production, representing >170 genes under various growth conditions (Msadek et al., 1990; Kobayashi, 2007; Verhamme et al., 2007; Murray et al., 2009). Unphosphorylated DegU appears to activate competence through the recruitment of ComK that acts as an anti-repressor for the transcriptional repressors Rok and CodY (Hamoen et al., 2000; Albano et al., 2005; Smits et al., 2007), whereas phosphorylated DegU directly recruits the RNA polymerase at promoter regions (Ogura et al., 2003; Verhamme et al., 2007; Murray et al., 2009). Unphosphorylated YycF (alias VicR) of *Streptococcus pneumoniae* directly inhibits transcription of the repressor gene *fabT*, and phosphorylation of YycF prevents binding to *fabB* involved in fatty acid metabolism, whereas it increases the binding affinity for the *pcsB*, *pspA*, *spr0096*, and *spr1875* promoters (Ng et al., 2005; Mohedano et al., 2016). Importantly, binding of YycF and YycF-P to these promoters occurs to the same sequence stretch as revealed by DNase I footprinting (Ng et al., 2005). Finally, whereas the dephosphomimetic mutant of CpxR from *Legionella pneumophila* is no longer capable of activating, it still functions as a repressor for other targets (Feldheim et al., 2016).

Experimental results on the effects of phosphorylated/unphosphorylated RRs and mutants of the conserved phosphoryl-receiver aspartate always have to be interpreted with caution since in many instances loss of function mutations in the HK are associated with enhanced expression of the cognate RR and its targets. Whereas this observation might at first sight suggest that the unphosphorylated form of the RR is the activating form, it was clearly demonstrated that transcription activation by *Streptomyces coelicolor* VanR in the absence of the cognate HK VanS requires *in vivo* phosphorylation of the RR by the cellular pool of acetylphosphate, which can act as a small phosphodonor for at least some RRs (Bouche et al., 1998; Da Re et al., 1999; Hutchings et al., 2006). In full agreement with this observation, the dephosphomimetic *vanR* mutant proved to be inactive in transcription stimulation. Though less thoroughly documented, a similar scenario was proposed for MtrAB of *S. coelicolor* (Som et al., 2017a) and *Streptomyces venezuelae*, which coordinates chloramphenicol production with sporulation (Som et al., 2017b). Along the same lines, overexpression of the dephosphomimetic form of the RR proved to be sufficient to complement a RR-deletion phenotype and enable target gene expression. This has been observed for PhoP, HrpY, and UhpA and may be explained by the residual activity of the unphosphorylated form when present at high, non-physiological concentrations and the dynamic nature of RRs that may lead to the coexistence of two sub-populations of which the relative abundance is influenced by phosphorylation (Webber and Kadner, 1997; Merighi et al., 2003; Lejona et al., 2004; Gao et al., 2007).

Footprinting and premodification binding interference experiments indicate that the 16-bp consensus core AgrR target site (AgrR box) consists of two hexameric half-sites organized in inverted repeat, separated by a 4-bp spacer (6-4-6) (Figure 6), which at least in some control regions (*argR*, *copA*_2_-*copR*_2_, *prsQ*_2_) is flanked on one or both sides by an imperfect direct repeat of the half site (Figure 5). Since purified His6- and MBP-tagged AgrR are essentially monomeric in solution, as are most RRs of the OmpR-family, the simultaneous binding to both halves of the palindromic target site implies that AgrR must at least form a symmetrical dimer on the DNA. Many OmpR-family members typically bind direct repeats, though they show high variability in sequence, number of direct repeats and spacer length (Martinez-Hackert and Stock, 1997), but some RRs bind sequences having dyad symmetry (Galperin, 2006, 2010). Phosphorylated *E. coli* OmpR, the archetype of the family, binds as a tandemly arranged dimer to a 20 bp target bearing two tandemly arranged 10 bp half-sites (Harrison-McMonagle et al., 1999). In contrast, CopR from *Pseudomonas syringae* and PcoR from *E. coli* plasmid pRJ1004, which show strong sequence similarity, bind an inverted repeat within a 16-bp stretch (Mills et al., 1993, 1994; Rouch and Brown, 1997), and whereas *E. coli* NarP only binds heptamers organized as inverted repeats with a 2-bp spacing (7-2-7), NarL binds a heptameric sequence found in various numbers and arrangements including direct and inverted repeats (Li et al., 1994; Darwin et al., 1996, 1997). Still other RRs require pronounced DNA distortions for high affinity binding (Maris et al., 2002; Feng et al., 2004).

Many RRs act as activators and are subjected to positive autoregulation, but some act as both activator and repressor. Here, we have focused on the characterization of genes and operons that are directly upregulated by AgrR in the silver-resistant mutant NA4S. Whether the AgrR regulon of *C. metallidurans* also comprises negatively regulated target genes is presently not known, however, the log2-fold change heatmap could indicate that such a gene set exists (Figure 1). The regulatory outcome of a RR may be correlated with the phosphorylation state and/or the position of the target with respect to the transcription initiation site, and the sequential occupation of multiple binding sites with a distinct affinity. As an example, the 19 bp consensus binding site of phosphorylated MtrA from *Corynebacterium glutamicum* consists of 8 bp direct repeats separated by 3 bp (8-3-8) and depending on the position of the binding site with respect to the transcription initiation site, MtrA-P may activate (*proP*, *betP*) or repress (*mepA*, *nlpC*) promoter activity (Brocker et al., 2011). Similarly, in the presence of zinc, *P. aeruginosa* CzcR exerts positive autoregulation and induces the expression of the *czcCBA* operon encoding an efflux pump, but represses the expression of porin encoding *oprD* and *phzA1* involved in pyocyanin production (Dieppois et al., 2012). *E. coli* OmpR that regulates the differential expression of porin genes acts as an activator for *ompC* and the small anti-sense RNA *micF*, but as a repressor for *ompF* under conditions of high osmolarity, as such OmpF is mainly produced under conditions of low osmolarity (Hall and Silhavy, 1981; Forst et al., 1989; Ishihama, 1993; Yoshida et al., 2006). PhoP is a dual regulator of numerous promoters of the PhoP regulon and exerts both positive and negative autoregulation (Kasahara et al., 1992; Kato et al., 1999; Yamamoto et al., 2002; Olivera et al., 2010).

The 16-bp core ArgR binding site is centered around position −43.5 with respect to the start of transcription in the own control region and slightly more upstream in the *prsQ*_2_, *copA*_2_, *czcR*_2_, *czcL* and *ompC*-like promoters (Figure 5). As all these promoters are upregulated in NA4S, it appears that unphosphorylated AgrR acts from this position as a Type II activator, directly stimulating RNA polymerase recruitment to promoters that generally harbor a reasonably good Pribnow box but no or a poorly conserved −35 promoter element (Figure 5), a characteristic of weak promoters requiring the help of an activator protein (Lee et al., 2012; Bervoets and Charlier, 2019). One exception to the proximity of the ArgR and RNA polymerase binding sites is the *czcI*_2_ control region, where the AgrR binding site is located much further upstream (centered around position −122.5). It is therefore plausible that AgrR-mediated activation of the *czcI*_2_ promoter may require the help of another DNA binding protein with DNA bending or bridging activity, as possess nucleoid associated proteins (NAPs). In addition, to complete the *czcI*_2_ results, *zntA*, which is involved in zinc homeostasis and upstream on the opposite strand of c*zcI*_2_, is not differentially expressed in the silver-resistant mutant NA4S in non-selective conditions.

Based on the identification and conservation of amino acid residues that contact the RNA polymerase α or σ^70^ subunit, RRs of the PhoB/OmpR family have been divided in two subgroups (Makino et al., 1996). Whereas the PhoB-like members contact σ^70^, OmpR-like members rather contact the α subunit. Though only one of the four residues of PhoB (W184) that directly contacts the α subunit is strictly conserved in AgrR, it nevertheless appears that the latter is more closely related to PhoB than OmpR in the protein-protein contact area and hence we hypothesize that AgrR might activate gene expression through direct interaction with the s subunit. However, this hypothesis is presently highly speculative and direct contacts between AgrR and one or more specific subunits of the RNA polymerase remain to be demonstrated.

## Data Availability Statement

The datasets presented in this study can be found in online repositories. The names of the repository/repositories and accession number(s) can be found at: https://www.ncbi.nlm.nih.gov/, PRJNA420641.

## Author Contributions

MA, RV, and DC contributed to conception and design of the study, and wrote the first draft of the manuscript. MA, AP, and DC performed the experimental work. KM, RV, and DC performed the data analyses. All authors contributed to manuscript revision read and approved the submitted version.

## Conflict of Interest

The authors declare that the research was conducted in the absence of any commercial or financial relationships that could be construed as a potential conflict of interest.
