# Supplementary material for: DNA-Binding and Transcription Activation by Unphosphorylated Response Regulator AgrR From Cupriavidus metallidurans Involved in Silver Resistance
Source: Front Microbiol. 2020 Jul 17;11:1635. doi: 10.3389/fmicb.2020.01635 (PMC7380067; doi:10.3389/fmicb.2020.01635)
Supplement: Supplementary file 1 [file Data_Sheet_1.DOCX]

Supplementary Material

# Supplementary Figures and Tables

## Supplementary Tables

| Supplementary Table 1. Primers and gBlocks gene fragments used in this study. | |
| --- | --- |
| Name | Sequence (5’ -> 3’)* |
| agrR_pMAL_FW | CATGAGAATTCATGAAGATCTTGGTAATCGAAGAT |
| agrR_pMAL_RV | CAGATAAGCTTTCAAGACTGTCGGTTCTCAAG |
| agrR_FW | AGCTGCATATGAAGATCTTGGTAATCGAAGA |
| agrR_RV | GCGAGAAGCTTAGACTGTCGGTTCTCAAGG |
| agrR_D51A_FW | GTCATTCTTGCCGTCATGCTGC |
| agrR_D51E_FW | GTCATTCTTGAGGTCATGCTGC |
| agrR_SDM_RV | CAGATCGTAGTCCCCGTCGAT |
|  |  |
| agrR_probe_FW | GCACAATCCACATGGTTCAC |
| agrR_probe_RV | GTTTTCATCCTCAGCTGCAC |
| copA2copR2_probe_FW | GTGTCCTCGTCAAACCAATG |
| copA2copR2_probe_RV | TTCTCATTGTGAAATCGCGC |
| czcI2_probe_FW | AGGACGTTATCAGGGGTGGT |
| czcI2_probe_RV | GGCGATTATAGGCGTTTAGC |
| czcLczcR2_probe_FW | TTGGTACCGGCCCATTATTC |
| czcLczcR2_probe_RV | TAGAACTGCCTGTGTTGAGC |
| prsQ1_probe_FW | TGAAGCCGATCCATGACATA |
| prsQ1_probe_RV | CATGATGGAATCCTCTTTCACA |
| prsQ2_probe_FW | GTTGTTTGGCGTGGATTTGT |
| prsQ2_probe_RV | ATGGGTTACGGGTTGTTCAC |
| v1_pm2526_probe_FW | AAGGTGAACTGGGTCGATTG |
| v1_pm2526_probe_RV | ATTGGAGCGGCGAGTATAGA |
|  |  |
| P*_prsQ2_* | TCGGAAGCTTCTCAGACTCGGACATGACTCATCGTGCGCGCCTGTCGACATGGCTGGACAGGCTGGAGAAAGGAAATTCTTGGGGGACGGCCCGCGCAGCGTGCATTGCGAACCAGATTGAGATTCTACAGGTTGTTTGGCGTGGATTTGTCGCCAAGGCCGAATTCATTGTCCGGTCGGCACCCTGTCCGGCAATCGTATCGCATTGCCTCGGACAAATGACATCAATGTCATTCAAGGGTCATGTTCGCGTCGGGACCCGCCCTCTAGACTGCATTCCAAGGTCGCGCAACGACCGATCAGACTCTCCAGCAAGCCGTGAACAACCCGTAACCCATATCACGAAGAGGATTCCCATATGCTGCAGGTCG |
| P*_prsQ2_*_(C>G,G>C)_ | TCGGAAGCTTCTCAGACTCGGACATGACTCATCGTGCGCGCCTGTCGACATGGCTGGACAGGCTGGAGAAAGGAAATTCTTGGGGGACGGCCCGCGCAGCGTGCATTGCGAACCAGATTGAGATTCTACAGGTTGTTTGGCGTGGATTTGTCGCCAAGGCCGAATTCATTGTCCGGTCGGCACCCTGTCCGGCAATCGTATCGCATTGCCTCGGACAAATGA**G**ATCAAT**C**TCATTCAAGGGTCATGTTCGCGTCGGGACCCGCCCTCTAGACTGCATTCCAAGGTCGCGCAACGACCGATCAGACTCTCCAGCAAGCCGTGAACAACCCGTAACCCATATCACGAAGAGGATTCCCATATGCTGCAGGTCG |
| P*_prsQ2_*_(C>T,G>A)_ | TCGGAAGCTTCTCAGACTCGGACATGACTCATCGTGCGCGCCTGTCGACATGGCTGGACAGGCTGGAGAAAGGAAATTCTTGGGGGACGGCCCGCGCAGCGTGCATTGCGAACCAGATTGAGATTCTACAGGTTGTTTGGCGTGGATTTGTCGCCAAGGCCGAATTCATTGTCCGGTCGGCACCCTGTCCGGCAATCGTATCGCATTGCCTCGGACAAATGA**T**ATCAAT**A**TCATTCAAGGGTCATGTTCGCGTCGGGACCCGCCCTCTAGACTGCATTCCAAGGTCGCGCAACGACCGATCAGACTCTCCAGCAAGCCGTGAACAACCCGTAACCCATATCACGAAGAGGATTCCCATATGCTGCAGGTCG |
| P*_prsQ2_*_(GT>CA)_ | TCGGAAGCTTCTCAGACTCGGACATGACTCATCGTGCGCGCCTGTCGACATGGCTGGACAGGCTGGAGAAAGGAAATTCTTGGGGGACGGCCCGCGCAGCGTGCATTGCGAACCAGATTGAGATTCTACAGGTTGTTTGGCGTGGATTTGTCGCCAAGGCCGAATTCATTGTCCGGTCGGCACCCTGTCCGGCAATCGTATCGCATTGCCTCGGACAAATGACATCAATGTCATTCAAGG**CA**CATGTTCGCGTCGGGACCCGCCCTCTAGACTGCATTCCAAGGTCGCGCAACGACCGATCAGACTCTCCAGCAAGCCGTGAACAACCCGTAACCCATATCACGAAGAGGATTCCCATATGCTGCAGGTCG |
| *Restriction sites are underlined, differences in gBlocks gene fragments are highlighted in bold and red. | |

| **Supplementary Table 2.** Summary of RNA-seq mapping statistics. | | | | | | | | | |
| --- | --- | --- | --- | --- | --- | --- | --- | --- | --- |
|  | NA4D*agrRS*_1 | NA4D*agrRS*_2 | NA4D*agrRS*_3 | NA4S_1 | NA4S_2 | NA4S_3 | NA4_1 | NA4_2 | NA4_3 |
| Total_fragments | 5500524 | 4703757 | 6477735 | 6491533 | 7877892 | 11704735 | 3576054 | 4643719 | 3888207 |
| Mapped_fragments | 5396152 | 4566601 | 6343562 | 6344585 | 7756837 | 11472888 | 3485282 | 4553527 | 3806754 |
| % Mapped_fragments | 98,10% | 97,08% | 97,93% | 97,74% | 98,46% | 98,02% | 97,46% | 98,06% | 97,91% |
| Uniquely_mapped_fragments | 5176325 | 4285326 | 5979392 | 6117284 | 7411564 | 11077645 | 3332207 | 4332360 | 3612545 |
| Multi_mapping_fragments | 219827 | 281275 | 364170 | 227301 | 345273 | 395243 | 153075 | 221167 | 194209 |
| Unmapped_fragments | 104372 | 137156 | 134173 | 146948 | 121055 | 231847 | 90772 | 90192 | 81453 |
| Properly_paired_fragments | 5258088 | 4473386 | 6175510 | 6185043 | 7594135 | 11214244 | 3414697 | 4478802 | 3713745 |
| Singleton_fragments | 105193 | 66762 | 121656 | 116248 | 118069 | 184609 | 50821 | 56951 | 72093 |
| More_than_one_chr_fragments | 6272 | 4514 | 8757 | 9468 | 12498 | 25517 | 4183 | 2608 | 3399 |
| Unexpected_strandness_fragments | 4152 | 3019 | 5408 | 3676 | 4600 | 8889 | 2649 | 1839 | 2284 |
| Unexpected_template_length | 13452 | 13265 | 22768 | 17446 | 19156 | 26826 | 7558 | 7452 | 7755 |
| Inversed_mapping | 8995 | 5655 | 9463 | 12704 | 8379 | 12803 | 5374 | 5875 | 7478 |
| Indels | 8323 | 7671 | 10184 | 10525 | 11965 | 17025 | 6125 | 7635 | 6455 |
| featureCounts assigned | 4505363 | 3836124 | 5239190 | 5324316 | 6578044 | 2908797 | 9524998 | 3806030 | 3217045 |
| %featureCounts assigned | 81,90% | 81,60% | 80,90% | 82,00% | 83,50% | 81,30% | 81,40% | 82,00% | 82,70% |
|  | | | | | | | | | |

| **Supplementary Table 3.** Log2-fold changes of differentially expressed genes in *C. metallidurans* NA4S versus NA4, NA4SΔ*agrRS* versus NA4S and NA4SΔ*agrRS* versus NA4 under non-selective growth conditions. | | | | | | | |
| --- | --- | --- | --- | --- | --- | --- | --- |
| Locus_tag^a^ | Product | NA4S  vs  NA4 | p | NA4SΔ*agrRS*  vs  NA4S | p | NA4SΔ*agrRS*  vs  NA4 | p |
| v1_pA0003 | conserved protein of unknown function | 3.46 | 0.00 | -3.79 | 0.00 | -0.34 | 1.00 |
| v1_pA0007 | protein of unknown function | 3.13 | 0.00 | -3.15 | 0.00 | -0.02 | 1.00 |
| v1_pA0008 | conserved protein of unknown function | 2.75 | 0.00 | -2.96 | 0.00 | -0.21 | 1.00 |
| v1_pA0019 | conserved protein of unknown function | 3.22 | 0.00 | -3.33 | 0.00 | -0.11 | 1.00 |
| v1_pA0028 | conserved protein of unknown function | 2.65 | 0.03 | -3.18 | 0.00 | -0.53 | 1.00 |
| v1_pA0033 | putative lipoprotein transmembrane | 3.29 | 0.00 | -3.50 | 0.00 | -0.20 | 1.00 |
| v1_pA0034 | conserved protein of unknown function | 3.27 | 0.00 | -3.69 | 0.00 | -0.42 | 1.00 |
| v1_pA0035 | conserved protein of unknown function | 3.94 | 0.00 | -4.14 | 0.00 | -0.20 | 1.00 |
| v1_pA0036 | conserved protein of unknown function | 3.12 | 0.00 | -3.23 | 0.00 | -0.11 | 1.00 |
| v1_pA0042 | conserved protein of unknown function | 1.65 | 0.06 | -1.84 | 0.01 | -0.19 | 1.00 |
| v1_pA0045 | conserved protein of unknown function | 3.16 | 0.00 | -3.42 | 0.00 | -0.26 | 1.00 |
| v1_pA0046 | protein of unknown function | 3.27 | 0.00 | -3.21 | 0.00 | 0.06 | 1.00 |
| v1_pA0047 | conserved protein of unknown function | 2.30 | 0.01 | -2.43 | 0.00 | -0.13 | 1.00 |
| v1_pA0048 | conserved protein of unknown function | 3.36 | 0.00 | -3.86 | 0.00 | -0.50 | 1.00 |
| v1_pA0049 | conserved protein of unknown function | 3.41 | 0.00 | -3.59 | 0.00 | -0.18 | 1.00 |
| v1_pA0050 | conserved protein of unknown function | 2.45 | 0.00 | -2.68 | 0.00 | -0.23 | 1.00 |
| v1_pA0053 | conserved protein of unknown function | 3.02 | 0.00 | -2.73 | 0.00 | 0.29 | 1.00 |
| v1_pA0054 | conserved protein of unknown function | 3.38 | 0.00 | -3.67 | 0.00 | -0.28 | 1.00 |
| v1_pA0097 | conserved protein of unknown function | 3.45 | 0.00 | -3.76 | 0.00 | -0.31 | 1.00 |
| v1_pA0138 | protein of unknown function | -2.16 | 0.43 | 3.22 | 0.01 | 1.05 | 1.00 |
| v1_pA0139 | protein of unknown function | -1.57 | 0.74 | 2.20 | 0.03 | 0.64 | 1.00 |
| v1_pA0148 | conserved protein of unknown function | 2.56 | 0.01 | -2.55 | 0.00 | 0.02 | 1.00 |
| v1_pA0149 | conserved protein of unknown function | 2.29 | 0.00 | -2.30 | 0.00 | -0.01 | 1.00 |
| v1_pA0159 | conserved protein of unknown function | 3.06 | 0.00 | -3.43 | 0.00 | -0.37 | 1.00 |
| v1_pA0160 | protein of unknown function | 2.96 | 0.00 | -3.06 | 0.00 | -0.09 | 1.00 |
| v1_pA0161 | conserved protein of unknown function | 2.77 | 0.00 | -3.02 | 0.00 | -0.25 | 1.00 |
| v1_pA0163 | protein of unknown function | 3.09 | 0.00 | -3.70 | 0.00 | -0.61 | 1.00 |
| v1_pA0165 | conserved protein of unknown function | 2.74 | 0.00 | -2.60 | 0.00 | 0.14 | 1.00 |
| v1_pA0166 | conserved protein of unknown function | 2.79 | 0.00 | -3.14 | 0.00 | -0.35 | 1.00 |
| v1_pA0167 | Endonuclease | 2.04 | 0.03 | -2.49 | 0.00 | -0.45 | 1.00 |
| v1_pA0169 | Conjugal transfer protein | 2.81 | 0.00 | -3.01 | 0.00 | -0.20 | 1.00 |
| v1_pA0173 | protein of unknown function | 3.45 | 0.00 | -3.92 | 0.00 | -0.47 | 1.00 |
| v1_pA0174 | conserved protein of unknown function | 2.82 | 0.00 | -2.73 | 0.00 | 0.09 | 1.00 |
| v1_pA0175 | conserved protein of unknown function | 3.31 | 0.00 | -3.89 | 0.00 | -0.58 | 1.00 |
| v1_pA0176 | conserved protein of unknown function | 3.79 | 0.00 | -4.08 | 0.00 | -0.29 | 1.00 |
| v1_pA0177 | conserved protein of unknown function | 3.81 | 0.00 | -4.30 | 0.00 | -0.49 | 1.00 |
| v1_pA0178 | SAM-dependent methyltransferase | 2.97 | 0.00 | -3.14 | 0.00 | -0.17 | 1.00 |
| v1_pA0181 | conserved protein of unknown function | 2.03 | 0.00 | -2.04 | 0.00 | -0.01 | 1.00 |
| v1_pA0182 | conserved protein of unknown function | 3.28 | 0.00 | -3.46 | 0.00 | -0.18 | 1.00 |
| v1_pA0184 | protein of unknown function | 2.38 | 0.00 | -2.51 | 0.00 | -0.13 | 1.00 |
| v1_pA0185 | conserved protein of unknown function | 2.70 | 0.00 | -3.19 | 0.00 | -0.49 | 1.00 |
| v1_pA0186 | conserved protein of unknown function | 2.72 | 0.00 | -3.05 | 0.00 | -0.33 | 1.00 |
| v1_pA0187 | protein of unknown function | 2.68 | 0.00 | -3.17 | 0.00 | -0.49 | 1.00 |
| v1_pA0188 | conserved protein of unknown function | 2.15 | 0.00 | -2.29 | 0.00 | -0.14 | 1.00 |
| v1_pA0195 | conserved protein of unknown function | 2.43 | 0.00 | -2.69 | 0.00 | -0.26 | 1.00 |
| v1_pA0234 | Heptosyltransferase-1 | 4.32 | 0.00 | -4.23 | 0.00 | 0.09 | 1.00 |
| v1_pA0250 | conserved membrane protein of unknown function | 1.53 | 0.07 | -1.71 | 0.01 | -0.18 | 1.00 |
| v1_pA0251 | Mechanosensitive ion channel protein MscS | 1.91 | 0.02 | -2.31 | 0.00 | -0.40 | 1.00 |
| v1_pA0252 | Helix-turn-helix transcriptiol regulator | 1.73 | 0.08 | -2.08 | 0.01 | -0.35 | 1.00 |
| v1_pA0253 | putative transmembrane protein | 3.38 | 0.00 | -3.47 | 0.00 | -0.09 | 1.00 |
| v1_pA0254 | GtrA family protein | 3.61 | 0.01 | -3.18 | 0.00 | 0.43 | 1.00 |
| v1_pA0255 | putative enzyme | 3.27 | 0.00 | -3.02 | 0.00 | 0.25 | 1.00 |
| v1_pA0258 | Cadmium-transporting ATPase | 3.37 | 0.00 | -3.31 | 0.00 | 0.06 | 1.00 |
| v1_pA0259 | protein of unknown function | 3.01 | 0.00 | -2.95 | 0.00 | 0.06 | 1.00 |
| v1_pA0260 | Flagellar basal body rod protein FlgB | 3.48 | 0.00 | -2.92 | 0.00 | 0.56 | 1.00 |
| v1_pA0262 | Porin | 6.39 | 0.00 | -6.53 | 0.00 | -0.14 | 1.00 |
| v1_pA0264 | conserved exported protein of unknown function | 8.01 | 0.00 | -7.84 | 0.00 | 0.17 | 1.00 |
| v1_pA0265 | protein of unknown function | 4.93 | 0.00 | -5.37 | 0.00 | -0.44 | 1.00 |
| v1_pA0266 | Heavy metal resistance protein CzcE | 4.01 | 0.00 | -3.91 | 0.00 | 0.10 | 1.00 |
| v1_pA0275 | protein of unknown function | 1.87 | 0.02 | -2.42 | 0.00 | -0.56 | 1.00 |
| v1_pA0288 | Alkaline phosphatase | 1.80 | 0.05 | -1.78 | 0.05 | 0.02 | 1.00 |
| v1_pA0289 | conserved exported protein of unknown function | 2.87 | 0.00 | -3.17 | 0.00 | -0.30 | 1.00 |
| v1_pA0291 | conserved protein of unknown function | 1.71 | 0.10 | -2.37 | 0.00 | -0.67 | 1.00 |
| v1_pA0302 | putative hemagglutinin-related transmembrane protein | 2.39 | 0.01 | -2.16 | 0.02 | 0.24 | 1.00 |
| v1_pA0309 | conserved protein of unknown function | 3.79 | 0.00 | -3.84 | 0.00 | -0.05 | 1.00 |
| v1_pA0311 | conserved protein of unknown function | 2.54 | 0.00 | -2.24 | 0.00 | 0.30 | 1.00 |
| v1_pA0312 | conserved protein of unknown function | 3.58 | 0.00 | -3.96 | 0.00 | -0.38 | 1.00 |
| v1_pA0313 | Cobalamin biosynthesis protein CobS | 3.45 | 0.00 | -3.66 | 0.00 | -0.20 | 1.00 |
| v1_pA0314 | conserved protein of unknown function | 2.86 | 0.00 | -3.05 | 0.00 | -0.19 | 1.00 |
| v1_pA0318 | conserved protein of unknown function | 2.93 | 0.00 | -3.06 | 0.00 | -0.13 | 1.00 |
| v1_pA0323 | conserved protein of unknown function | 3.23 | 0.00 | -3.27 | 0.00 | -0.04 | 1.00 |
| v1_pA0324 | Vitamin B12-dependent ribonucleotide reductase | 2.16 | 0.00 | -2.30 | 0.00 | -0.14 | 1.00 |
| v1_pA0328 | Type II secretion system protein E | 2.29 | 0.02 | -1.91 | 0.10 | 0.38 | 1.00 |
| v1_pA0337 | conserved membrane protein of unknown function | 3.94 | 0.01 | -3.31 | 0.00 | 0.63 | 1.00 |
| v1_pA0338 | Membrane lipoprotein lipid attachment site | 3.85 | 0.00 | -3.93 | 0.00 | -0.08 | 1.00 |
| v1_pA0339 | TraB pilus assembly family protein | 2.90 | 0.01 | -2.39 | 0.02 | 0.50 | 1.00 |
| v1_pA0340 | putative transfer protein TraK | 2.85 | 0.01 | -2.74 | 0.01 | 0.11 | 1.00 |
| v1_pA0344 | conserved protein of unknown function | 3.02 | 0.00 | -2.99 | 0.00 | 0.02 | 1.00 |
| v1_pA0350 | conserved protein of unknown function | 1.83 | 0.07 | -2.02 | 0.02 | -0.19 | 1.00 |
| v1_pA0356 | conserved protein of unknown function | 2.80 | 0.00 | -3.22 | 0.00 | -0.42 | 1.00 |
| v1_pA0358 | conserved protein of unknown function | 3.11 | 0.00 | -3.37 | 0.00 | -0.26 | 1.00 |
| v1_pA0359 | conserved protein of unknown function | 3.22 | 0.00 | -3.43 | 0.00 | -0.21 | 1.00 |
| v1_pA0360 | conserved exported protein of unknown function | 2.10 | 0.01 | -1.76 | 0.02 | 0.33 | 1.00 |
| v1_pA0361 | Sex pilus assembly and mating pair formation protein | 2.15 | 0.01 | -1.77 | 0.04 | 0.37 | 1.00 |
| v1_pA0363 | Sex pilus assembly and synthesis protein | 1.95 | 0.10 | -2.24 | 0.02 | -0.29 | 1.00 |
| v1_pA0364 | Thiol:disulfide interchange protein | 2.15 | 0.05 | -2.10 | 0.03 | 0.04 | 1.00 |
| v1_pA0367 | C-5 cytosine-specific D methylase | 2.74 | 0.00 | -3.13 | 0.00 | -0.39 | 1.00 |
| v1_pA0369 | conserved protein of unknown function | 2.17 | 0.00 | -2.42 | 0.00 | -0.25 | 1.00 |
| v1_pA0370 | conserved protein of unknown function | 2.38 | 0.02 | -2.72 | 0.00 | -0.34 | 1.00 |
| v1_pA0375 | protein of unknown function | 1.43 | 0.80 | -1.96 | 0.03 | -0.53 | 1.00 |
| v1_pA0401 | putative cysteine peptidase | 2.57 | 0.00 | -2.76 | 0.00 | -0.19 | 1.00 |
| v1_pA0402 | Protein TraB (fragment) | 2.44 | 0.01 | -2.83 | 0.00 | -0.39 | 1.00 |
| v1_pA0403 | conserved protein of unknown function | 2.24 | 0.00 | -2.46 | 0.00 | -0.22 | 1.00 |
| v1_pA0404 | conserved protein of unknown function | 2.50 | 0.00 | -2.68 | 0.00 | -0.18 | 1.00 |
| v1_pA0405 | conserved protein of unknown function | 3.54 | 0.00 | -3.91 | 0.00 | -0.37 | 1.00 |
| v1_pA0406 | conserved protein of unknown function | 2.79 | 0.00 | -3.21 | 0.00 | -0.42 | 1.00 |
| v1_pA0407 | PAPS reductase/FAD synthetase | 2.37 | 0.00 | -2.65 | 0.00 | -0.28 | 1.00 |
| v1_pA0409 | conserved protein of unknown function | 2.80 | 0.00 | -2.90 | 0.00 | -0.11 | 1.00 |
| v1_pA0411 | conserved exported protein of unknown function | 2.60 | 0.00 | -2.93 | 0.00 | -0.33 | 1.00 |
| v1_pA0412 | conserved protein of unknown function | 2.62 | 0.00 | -2.90 | 0.00 | -0.28 | 1.00 |
| v1_pA0413 | protein of unknown function | 2.74 | 0.00 | -3.22 | 0.00 | -0.48 | 1.00 |
| v1_pA0414 | putative N-6 D Methylase | 2.92 | 0.00 | -2.94 | 0.00 | -0.02 | 1.00 |
| v1_pA0415 | conserved protein of unknown function | 2.57 | 0.00 | -2.50 | 0.00 | 0.07 | 1.00 |
| v1_pB0042 | conserved protein of unknown function | 1.37 | 0.95 | -2.05 | 0.01 | -0.69 | 1.00 |
| v1_pB0052 | conserved protein of unknown function | 2.76 | 0.02 | -2.59 | 0.01 | 0.17 | 1.00 |
| v1_pB0053 | Carbamoylphosphate synthase large subunit | 2.93 | 0.00 | -2.96 | 0.00 | -0.04 | 1.00 |
| v1_pB0054 | conserved protein of unknown function | 2.61 | 0.00 | -2.37 | 0.00 | 0.23 | 1.00 |
| v1_pB0055 | conserved protein of unknown function | 2.39 | 0.01 | -2.24 | 0.01 | 0.15 | 1.00 |
| v1_pB0060 | conserved exported protein of unknown function | 2.35 | 0.00 | -2.84 | 0.00 | -0.49 | 1.00 |
| v1_pB0061 | conserved protein of unknown function | 1.76 | 0.14 | -2.04 | 0.01 | -0.27 | 1.00 |
| v1_pB0062 | conserved protein of unknown function | 0.97 | 1.00 | -1.54 | 0.04 | -0.58 | 1.00 |
| v1_pB0064 | conserved protein of unknown function | 2.63 | 0.01 | -2.49 | 0.00 | 0.14 | 1.00 |
| v1_pB0066 | conserved protein of unknown function | 3.32 | 0.00 | -2.98 | 0.00 | 0.35 | 1.00 |
| v1_pB0073 | protein of unknown function | 4.44 | 0.00 | -4.76 | 0.00 | -0.32 | 1.00 |
| v1_pB0074 | conserved protein of unknown function | 2.71 | 0.00 | -2.16 | 0.01 | 0.56 | 1.00 |
| v1_pB0077 | conserved protein of unknown function | 2.09 | 0.07 | -2.06 | 0.03 | 0.03 | 1.00 |
| v1_pB0080 | conserved protein of unknown function | 2.42 | 0.05 | -2.16 | 0.04 | 0.26 | 1.00 |
| v1_pB0114 | conserved exported protein of unknown function | 6.83 | 0.00 | -7.11 | 0.00 | -0.27 | 1.00 |
| v1_pB0115 | putative cadmium-transporting ATPase | 4.11 | 0.00 | -4.33 | 0.00 | -0.22 | 1.00 |
| v1_pB0138 | Flagellar basal body rod protein FlgB | 3.78 | 0.00 | -3.80 | 0.00 | -0.01 | 1.00 |
| v1_pB0142 | protein of unknown function | 3.78 | 0.00 | -3.99 | 0.00 | -0.20 | 1.00 |
| v1_pB0209 | conserved protein of unknown function | 1.88 | 0.04 | -2.27 | 0.00 | -0.38 | 1.00 |
| v1_pB0212 | transposase | 1.81 | 0.01 | -2.12 | 0.00 | -0.31 | 1.00 |
| v1_pB0235 | conserved protein of unknown function | 2.05 | 0.00 | -2.04 | 0.00 | 0.01 | 1.00 |
| v1_pB0244 | Conjugal transfer protein | 2.17 | 0.10 | -2.27 | 0.03 | -0.10 | 1.00 |
| v1_pB0245 | conserved membrane protein of unknown function | 2.50 | 0.03 | -1.90 | 0.07 | 0.60 | 1.00 |
| v1_pB0249 | conserved exported protein of unknown function | 2.39 | 0.02 | -1.45 | 0.41 | 0.95 | 1.00 |
| v1_pB0260 | Conjugal transfer protein | 2.10 | 0.00 | -2.25 | 0.00 | -0.15 | 1.00 |
| v1_pC0002 | Pilus assembly protein PilX | 3.06 | 0.00 | -3.05 | 0.00 | 0.01 | 1.00 |
| v1_pC0004 | Type IV pilus biosynthesis protein PilQ | 1.78 | 0.04 | -1.55 | 0.15 | 0.23 | 1.00 |
| v1_pC0005 | putative Type IV pilus biogenesis protein PilP | 2.16 | 0.02 | -1.79 | 0.07 | 0.37 | 1.00 |
| v1_pC0007 | Type IV pilus biogenesis protein, PilN | 2.34 | 0.00 | -2.34 | 0.00 | 0.00 | 1.00 |
| v1_pC0010 | conserved protein of unknown function | 2.64 | 0.00 | -2.42 | 0.00 | 0.21 | 1.00 |
| v1_pC0015 | Type IV secretion protein DotD | 2.28 | 0.00 | -2.45 | 0.00 | -0.17 | 1.00 |
| v1_pC0036 | conserved protein of unknown function | 1.70 | 0.07 | -1.89 | 0.01 | -0.19 | 1.00 |
| v1_pC0037 | Type IV secretion protein IcmL | 2.51 | 0.00 | -2.33 | 0.00 | 0.18 | 1.00 |
| v1_pC0038 | Type IV secretion protein IcmK | 1.84 | 0.03 | -1.62 | 0.07 | 0.22 | 1.00 |
| v1_pC0039 | Type IV secretion protein DotG | 2.39 | 0.00 | -2.22 | 0.00 | 0.17 | 1.00 |
| v1_pC0040 | conserved protein of unknown function | 2.17 | 0.00 | -2.06 | 0.00 | 0.11 | 1.00 |
| v1_pC0041 | Type IV secretion protein IcmC | 2.49 | 0.02 | -2.39 | 0.01 | 0.10 | 1.00 |
| v1_pC0042 | conserved membrane protein of unknown function | 2.78 | 0.00 | -2.57 | 0.00 | 0.22 | 1.00 |
| v1_pC0043 | Type IV secretion protein IcmJ | 2.21 | 0.02 | -2.16 | 0.01 | 0.05 | 1.00 |
| v1_pC0048 | conserved exported protein of unknown function | 2.00 | 0.04 | -1.71 | 0.11 | 0.30 | 1.00 |
| v1_pC0058 | D helicase | 1.83 | 0.03 | -2.03 | 0.01 | -0.20 | 1.00 |
| v1_pC0059 | conserved protein of unknown function | 2.84 | 0.00 | -2.67 | 0.00 | 0.17 | 1.00 |
| v1_pC0065 | conserved protein of unknown function | 1.81 | 0.10 | -1.92 | 0.03 | -0.11 | 1.00 |
| v1_pC0067 | Putative membrane protein (modular protein) | 2.17 | 0.03 | -1.95 | 0.03 | 0.22 | 1.00 |
| v1_pC0073 | protein of unknown function | 1.48 | 0.18 | -1.71 | 0.02 | -0.23 | 1.00 |
| v1_pC0118 | conserved protein of unknown function | 1.79 | 0.35 | -2.49 | 0.01 | -0.69 | 1.00 |
| v1_pC0124 | Single-stranded D-binding protein | 2.42 | 0.00 | -2.72 | 0.00 | -0.30 | 1.00 |
| v1_pC0138 | conserved exported protein of unknown function | 1.36 | 0.68 | -1.93 | 0.01 | -0.56 | 1.00 |
| v1_pC0145 | conserved protein of unknown function | 2.52 | 0.00 | -2.47 | 0.00 | 0.05 | 1.00 |
| v1_pC0146 | transposase | 1.99 | 0.01 | -1.50 | 0.15 | 0.49 | 1.00 |
| v1_pC0147 | protein of unknown function | 3.37 | 0.01 | -3.11 | 0.00 | 0.26 | 1.00 |
| v1_pC0148 | protein of unknown function | 2.01 | 0.01 | -2.34 | 0.00 | -0.33 | 1.00 |
| v1_pC0149 | D primase TraC (fragment) | 2.37 | 0.00 | -2.65 | 0.00 | -0.28 | 1.00 |
| v1_pC0150 | conserved protein of unknown function | 1.24 | 1.00 | -1.90 | 0.05 | -0.65 | 1.00 |
| v1_pC0151 | protein of unknown function | 1.54 | 0.35 | -2.05 | 0.01 | -0.50 | 1.00 |
| v1_pC0153 | Thiol:disulfide interchange protein | 1.73 | 0.02 | -1.92 | 0.00 | -0.20 | 1.00 |
| v1_pC0156 | conserved protein of unknown function | 2.05 | 0.03 | -2.08 | 0.01 | -0.03 | 1.00 |
| v1_pC0161 | conserved protein of unknown function | 2.29 | 0.03 | -2.06 | 0.04 | 0.23 | 1.00 |
| v1_pC0167 | conserved protein of unknown function | 1.86 | 0.01 | -1.39 | 0.29 | 0.47 | 1.00 |
| v1_pC0187 | conserved exported protein of unknown function | 1.98 | 0.01 | -2.11 | 0.00 | -0.13 | 1.00 |
| v1_pC0188 | conserved protein of unknown function | 1.59 | 0.04 | -1.53 | 0.05 | 0.06 | 1.00 |
| v1_pC0190 | conserved exported protein of unknown function | 1.91 | 0.09 | -2.10 | 0.02 | -0.19 | 1.00 |
| v1_pC0192 | conserved protein of unknown function | 2.01 | 0.04 | -1.99 | 0.03 | 0.02 | 1.00 |
| v1_pC0200 | conserved protein of unknown function | 1.22 | 1.00 | -1.82 | 0.02 | -0.60 | 1.00 |
| v1_pD0077 | conserved protein of unknown function | -2.37 | 0.01 | 2.37 | 0.01 | -0.01 | 1.00 |
| v1_pD0078 | D primase | -2.56 | 0.01 | 2.68 | 0.01 | 0.12 | 1.00 |
| v1_pm0032 | Acyl-homoserine lactone acylase PvdQ | -1.47 | 0.46 | 1.84 | 0.03 | 0.37 | 1.00 |
| v1_pm0074 | protein of unknown function | -2.09 | 0.92 | 3.24 | 0.03 | 1.14 | 1.00 |
| v1_pm0194 | Type II secretory pathway, gspf-related transmembrane protein | -1.28 | 1.00 | 2.35 | 0.02 | 1.07 | 1.00 |
| v1_pm0203 | conserved protein of unknown function | -1.78 | 1.00 | 3.11 | 0.01 | 1.32 | 1.00 |
| v1_pm0360 | conserved protein of unknown function | -1.91 | 0.00 | 1.93 | 0.00 | 0.02 | 1.00 |
| v1_pm0417 | CopM involved in Cu(II)/Cu(I) resistance | 4.49 | 0.00 | -4.91 | 0.00 | -0.41 | 1.00 |
| v1_pm0418 | Copper resistance protein K | 5.60 | 0.00 | -6.35 | 0.00 | -0.75 | 1.00 |
| v1_pm0422 | Copper resistance protein A | 3.56 | 0.00 | -3.67 | 0.00 | -0.11 | 1.00 |
| v1_pm0423 | Copper resistance protein B precursor | 3.72 | 0.00 | -3.55 | 0.00 | 0.17 | 1.00 |
| v1_pm0424 | Copper resistance protein C | 4.63 | 0.00 | -4.54 | 0.00 | 0.09 | 1.00 |
| v1_pm0425 | Copper resistance protein D | 3.83 | 0.00 | -4.06 | 0.00 | -0.24 | 1.00 |
| v1_pm0426 | Copper-binding protein | 2.69 | 0.00 | -2.81 | 0.00 | -0.12 | 1.00 |
| v1_pm0427 | CytoCHR1e c | 3.63 | 0.00 | -3.81 | 0.00 | -0.17 | 1.00 |
| v1_pm0428 | CopG family transcriptiol regulator | 4.30 | 0.00 | -4.59 | 0.00 | -0.29 | 1.00 |
| v1_pm0430 | conserved exported protein of unknown function | 4.08 | 0.00 | -4.28 | 0.00 | -0.20 | 1.00 |
| v1_pm0431 | CopH involved in Cu(II)/Cu(I) resistance (CzcE-like protein) up with Mn | 2.85 | 0.00 | -2.98 | 0.00 | -0.13 | 1.00 |
| v1_pm0433 | conserved protein of unknown function | 1.37 | 0.54 | -1.68 | 0.04 | -0.32 | 1.00 |
| v1_pm0434 | Outer membrane protein | 4.99 | 0.00 | -5.43 | 0.00 | -0.45 | 1.00 |
| v1_pm0438 | Methyltransferase domain-containing protein | 1.79 | 0.02 | -2.05 | 0.00 | -0.26 | 1.00 |
| v1_pm0439 | Phosphoesterase | 2.49 | 0.00 | -2.70 | 0.00 | -0.21 | 1.00 |
| v1_pm0447 | conserved exported protein of unknown function | 7.08 | 0.00 | -7.36 | 0.00 | -0.28 | 1.00 |
| v1_pm0449 | protein of unknown function | 1.15 | 1.00 | -1.65 | 0.03 | -0.50 | 1.00 |
| v1_pm0450 | protein of unknown function | 1.40 | 0.43 | -1.74 | 0.03 | -0.34 | 1.00 |
| v1_pm0512 | conserved protein of unknown function | 1.61 | 0.21 | -2.52 | 0.00 | -0.92 | 1.00 |
| v1_pm0515 | conserved protein of unknown function | 1.34 | 1.00 | -2.43 | 0.04 | -1.09 | 1.00 |
| v1_pm0516 | transposase | 1.94 | 0.01 | -2.22 | 0.00 | -0.28 | 1.00 |
| v1_pm0517 | conserved protein of unknown function | 1.48 | 0.64 | -2.39 | 0.00 | -0.91 | 1.00 |
| v1_pm0518 | transposase | 1.96 | 0.00 | -2.23 | 0.00 | -0.28 | 1.00 |
| v1_pm0539 | conserved protein of unknown function | -1.92 | 0.60 | 2.87 | 0.02 | 0.95 | 1.00 |
| v1_pm0540 | conserved protein of unknown function | -3.47 | 0.00 | 3.57 | 0.00 | 0.10 | 1.00 |
| v1_pm0541 | Polyamine aminopropyltransferase | -2.78 | 0.01 | 3.26 | 0.00 | 0.48 | 1.00 |
| v1_pm0542 | Nonspecific acid phosphatase pD-like protein | -3.82 | 0.00 | 3.95 | 0.00 | 0.13 | 1.00 |
| v1_pm0543 | Serine/threonine-protein kise pkn1 | -3.16 | 0.00 | 3.50 | 0.00 | 0.34 | 1.00 |
| v1_pm0545 | conserved exported protein of unknown function | -2.57 | 0.30 | 3.08 | 0.05 | 0.51 | 1.00 |
| v1_pm0546 | conserved protein of unknown function | -2.68 | 0.03 | 2.87 | 0.01 | 0.19 | 1.00 |
| v1_pm0550 | conserved exported protein of unknown function | -2.92 | 0.00 | 2.83 | 0.00 | -0.09 | 1.00 |
| v1_pm0591 | dipeptide transporter_ membrane component of ABC superfamily | -2.11 | 0.07 | 2.29 | 0.03 | 0.18 | 1.00 |
| v1_pm0592 | Oligopeptide transport system permease protein AppC | -2.29 | 0.07 | 2.82 | 0.01 | 0.53 | 1.00 |
| v1_pm0593 | Heme-binding protein A | -2.17 | 0.01 | 2.26 | 0.01 | 0.10 | 1.00 |
| v1_pm0594 | ATP-binding component of ABC superfamily | -2.17 | 0.04 | 2.20 | 0.03 | 0.03 | 1.00 |
| v1_pm0595 | ATP-binding component of ABC superfamily | -1.43 | 0.69 | 1.88 | 0.03 | 0.45 | 1.00 |
| v1_pm0610 | Cobalt-zinc-cadmium resistance protein CzcI | 2.19 | 0.00 | -2.37 | 0.00 | -0.18 | 1.00 |
| v1_pm0615 | putative 3-demethylubiquinone-9 3-methyltransferase | 1.31 | 0.64 | -1.61 | 0.04 | -0.30 | 1.00 |
| v1_pm0616 | Sensor protein | 3.87 | 0.00 | -4.07 | 0.00 | -0.20 | 1.00 |
| v1_pm0617 | response regulator in two-component regulatory system | 2.66 | 0.00 | -2.79 | 0.00 | -0.13 | 1.00 |
| v1_pm0618 | conserved exported protein of unknown function | 2.94 | 0.00 | -2.87 | 0.00 | 0.07 | 1.00 |
| v1_pm0620 | Protein involved in acute metal response (CopQ/CzcJ/MmrQ/MmtQlike) | 8.99 | 0.00 | -8.39 | 0.00 | 0.60 | 1.00 |
| v1_pm0661 | conserved exported protein of unknown function | -1.49 | 0.85 | 2.23 | 0.01 | 0.74 | 1.00 |
| v1_pm0865 | conserved exported protein of unknown function | 1.82 | 0.04 | -2.12 | 0.00 | -0.29 | 1.00 |
| v1_pm0910 | conserved protein of unknown function | -2.12 | 0.54 | 2.92 | 0.03 | 0.80 | 1.00 |
| v1_pm0912 | Dihydrolipoyl dehydrogese | -1.67 | 0.02 | 1.84 | 0.00 | 0.17 | 1.00 |
| v1_pm0937 | Non-hemolytic phospholipase C | -2.21 | 0.22 | 3.15 | 0.01 | 0.94 | 1.00 |
| v1_pm1000 | 1-Cysteine peroxiredoxin (Thiol peroxidase) | -1.88 | 0.03 | 1.95 | 0.02 | 0.07 | 1.00 |
| v1_pm1006 | conserved protein of unknown function | 2.33 | 0.00 | -2.28 | 0.00 | 0.05 | 1.00 |
| v1_pm1042 | Paraquat-inducible protein A | -1.30 | 1.00 | 1.88 | 0.02 | 0.58 | 1.00 |
| v1_pm1046 | Alkaline phosphatase H | -1.69 | 0.09 | 2.10 | 0.01 | 0.41 | 1.00 |
| v1_pm1069 | Chaperone pD | -2.43 | 0.00 | 2.66 | 0.00 | 0.23 | 1.00 |
| v1_pm1070 | nitrate reductase, periplasmic, large subunit | -2.38 | 0.00 | 2.90 | 0.00 | 0.52 | 1.00 |
| v1_pm1071 | Periplasmic nitrate reductase, electron transfer subunit | -2.66 | 0.00 | 3.31 | 0.00 | 0.65 | 1.00 |
| v1_pm1072 | nitrate reductase, cytoCHR1e c-type,periplasmic | -2.13 | 0.00 | 2.48 | 0.00 | 0.35 | 1.00 |
| v1_pm1073 | ATP-binding component of ABC superfamily | -2.71 | 0.11 | 3.36 | 0.01 | 0.65 | 1.00 |
| v1_pm1074 | membrane component of ABC superfamily | -2.46 | 0.53 | 3.35 | 0.03 | 0.89 | 1.00 |
| v1_pm1075 | Heme exporter protein C | -2.64 | 0.37 | 3.45 | 0.03 | 0.81 | 1.00 |
| v1_pm1077 | heme lyase, CcmF subunit | -1.75 | 1.00 | 2.86 | 0.03 | 1.11 | 1.00 |
| v1_pm1181 | conserved exported protein of unknown function | -1.40 | 0.43 | 1.69 | 0.04 | 0.29 | 1.00 |
| v1_pm1185 | conserved protein of unknown function | -2.29 | 0.00 | 2.04 | 0.00 | -0.25 | 1.00 |
| v1_pm1232 | Esterase | -2.01 | 0.02 | 2.26 | 0.00 | 0.25 | 1.00 |
| v1_pm1325 | Transcriptiol regulator, LuxR family | -2.15 | 0.00 | 2.05 | 0.00 | -0.10 | 1.00 |
| v1_pm1358 | protein of unknown function | -2.20 | 0.04 | 2.50 | 0.01 | 0.30 | 1.00 |
| v1_pm1394 | Calcium-binding EF-hand protein | -2.67 | 0.00 | 2.75 | 0.00 | 0.08 | 1.00 |
| v1_pm1461 | Copper resistance protein A | 2.94 | 0.00 | -3.01 | 0.00 | -0.06 | 1.00 |
| v1_pm1462 | Copper resistance protein B precursor | 2.51 | 0.00 | -2.39 | 0.00 | 0.11 | 1.00 |
| v1_pm1463 | Copper resistance protein C | 2.78 | 0.00 | -3.08 | 0.00 | -0.31 | 1.00 |
| v1_pm1464 | Copper resistance protein D | 2.91 | 0.00 | -2.31 | 0.00 | 0.60 | 1.00 |
| v1_pm1469 | conserved protein of unknown function | -2.80 | 0.04 | 1.57 | 1.00 | -1.23 | 1.00 |
| v1_pm1492 | Flagellar hook-associated protein 2 | 1.51 | 0.09 | -1.81 | 0.01 | -0.30 | 1.00 |
| v1_pm1526 | Methyl-accepting chemotaxis protein I | 1.93 | 0.00 | -1.75 | 0.01 | 0.18 | 1.00 |
| v1_pm1528 | cytoCHR1e o ubiquinol oxidase subunit III | -1.74 | 0.10 | 1.81 | 0.05 | 0.06 | 1.00 |
| v1_pm1529 | cytoCHR1e o ubiquinol oxidase subunit I | -1.46 | 0.24 | 1.65 | 0.05 | 0.18 | 1.00 |
| v1_pm1638 | Multidrug transporter | -2.17 | 0.04 | 1.78 | 0.20 | -0.39 | 1.00 |
| v1_pm1639 | Cobalt-zinc-cadmium resistance protein CzcB | -2.56 | 0.00 | 2.34 | 0.00 | -0.22 | 1.00 |
| v1_pm1640 | Cobalt-zinc-cadmium resistance protein CzcA | -2.26 | 0.00 | 2.23 | 0.00 | -0.03 | 1.00 |
| v1_pm1767 | putative tight adherence (TadE/G) protein | -2.18 | 0.11 | 2.61 | 0.02 | 0.43 | 1.00 |
| v1_pm1852 | Flagellar protein FlgN | 1.23 | 0.94 | -1.50 | 0.05 | -0.28 | 1.00 |
| v1_pm1924 | Arylsulfatase-like enzyme | -3.20 | 0.00 | 3.55 | 0.00 | 0.34 | 1.00 |
| v1_pm1939 | putative alkyl sulfatase | -2.48 | 0.00 | 2.87 | 0.00 | 0.38 | 1.00 |
| v1_pm2031 | Response regulator | -2.36 | 0.10 | 2.49 | 0.04 | 0.14 | 1.00 |
| v1_pm2032 | conserved protein of unknown function | -2.03 | 0.09 | 2.22 | 0.02 | 0.19 | 1.00 |
| v1_pm2033 | Enterobactin exporter EntS | -2.34 | 0.19 | 2.70 | 0.04 | 0.36 | 1.00 |
| v1_pm2034 | conserved membrane protein of unknown function | -2.63 | 0.13 | 3.04 | 0.03 | 0.41 | 1.00 |
| v1_pm2035 | Amidohydrolase | -2.31 | 0.01 | 2.93 | 0.00 | 0.62 | 1.00 |
| v1_pm2036 | Non-heme haloperoxidase | -3.35 | 0.00 | 3.94 | 0.00 | 0.59 | 1.00 |
| v1_pm2037 | Hydrolase | -3.14 | 0.00 | 3.39 | 0.00 | 0.25 | 1.00 |
| v1_pm2040 | conserved protein of unknown function | 4.48 | 0.00 | -4.39 | 0.00 | 0.10 | 1.00 |
| v1_pm2111 | flagellar filament structural protein (flagellin) | 1.77 | 0.02 | -1.72 | 0.03 | 0.05 | 1.00 |
| v1_pm2160 | protein of unknown function | 6.39 | 0.00 | -6.31 | 0.00 | 0.08 | 1.00 |
| v1_pm2161 | conserved exported protein of unknown function | 4.60 | 0.00 | -4.91 | 0.00 | -0.31 | 1.00 |
| v1_pm2162 | Flagellar basal body rod protein FlgB | 3.17 | 0.00 | -3.08 | 0.00 | 0.10 | 1.00 |
| v1_pm2164 | conserved protein of unknown function | 1.82 | 0.01 | -2.08 | 0.00 | -0.25 | 1.00 |
| v1_pm2165 | Diguanylate cyclase/phosphodiesterase | 1.59 | 0.03 | -1.70 | 0.01 | -0.11 | 1.00 |
| v1_pm2166 | MFS transporter | 2.32 | 0.00 | -2.47 | 0.00 | -0.15 | 1.00 |
| v1_pm2167 | conserved protein of unknown function | 2.68 | 0.00 | -2.97 | 0.00 | -0.28 | 1.00 |
| v1_pm2175 | conserved exported protein of unknown function | 2.68 | 0.00 | -2.39 | 0.00 | 0.29 | 1.00 |
| v1_pm2176 | Methyltransferase domain-containing protein | 2.51 | 0.00 | -2.40 | 0.00 | 0.12 | 1.00 |
| v1_pm2180 | Porin | 4.91 | 0.00 | -5.20 | 0.00 | -0.29 | 1.00 |
| v1_pm2183 | protein of unknown function | 3.11 | 0.02 | -2.80 | 0.02 | 0.31 | 1.00 |
| v1_pm2189 | Tyrosine recombise XerD | 2.92 | 0.00 | -2.84 | 0.00 | 0.08 | 1.00 |
| v1_pm2190 | TolC family protein | 4.23 | 0.00 | -4.07 | 0.00 | 0.16 | 1.00 |
| v1_pm2191 | conserved protein of unknown function | 2.17 | 0.00 | -2.48 | 0.00 | -0.31 | 1.00 |
| v1_pm2192 | Ferric uptake regulation protein | 2.03 | 0.01 | -2.43 | 0.00 | -0.40 | 1.00 |
| v1_pm2193 | bifunctiol GDP-fucose synthetase | 1.90 | 0.01 | -1.84 | 0.01 | 0.06 | 1.00 |
| v1_pm2194 | NUDIX domain-containing protein | 1.64 | 0.02 | -1.73 | 0.00 | -0.09 | 1.00 |
| v1_pm2195 | putative glycosyltransferase | 4.15 | 0.00 | -4.42 | 0.00 | -0.26 | 1.00 |
| v1_pm2196 | conserved exported protein of unknown function | 7.31 | 0.00 | -7.71 | 0.00 | -0.41 | 1.00 |
| v1_pm2197 | Cu(II) resistance protein CopE (fragment) | 1.27 | 1.00 | -1.75 | 0.02 | -0.48 | 1.00 |
| v1_pm2198 | CopH protein | 3.78 | 0.00 | -3.57 | 0.00 | 0.22 | 1.00 |
| v1_pm2199 | conserved exported protein of unknown function | 8.15 | 0.00 | -7.49 | 0.00 | 0.66 | 1.00 |
| v1_pm2201 | conserved protein of unknown function | 3.38 | 0.00 | -2.38 | 0.01 | 0.99 | 1.00 |
| v1_pm2202 | Copper-transporting P-type ATPase | 2.11 | 0.00 | -1.24 | 0.96 | 0.87 | 1.00 |
| v1_pm2203 | CopG involved in survival in presence of high bioavailable Cu(II) | 2.50 | 0.01 | -1.87 | 0.09 | 0.63 | 1.00 |
| v1_pm2204 | CopJ protein | 2.08 | 0.01 | -1.44 | 0.40 | 0.65 | 1.00 |
| v1_pm2205 | Copper-binding protein | 2.96 | 0.00 | -2.37 | 0.00 | 0.59 | 1.00 |
| v1_pm2206 | Copper resistance protein D | 4.04 | 0.00 | -4.03 | 0.00 | 0.01 | 1.00 |
| v1_pm2207 | Copper resistance protein C | 3.86 | 0.00 | -3.99 | 0.00 | -0.13 | 1.00 |
| v1_pm2208 | Copper resistance protein B | 2.59 | 0.00 | -2.58 | 0.00 | 0.01 | 1.00 |
| v1_pm2209 | Copper resistance protein A | 2.99 | 0.00 | -3.26 | 0.00 | -0.26 | 1.00 |
| v1_pm2213 | Copper resistance protein K | 3.30 | 0.00 | -4.11 | 0.00 | -0.81 | 1.00 |
| v1_pm2214 | Cu(II)/Cu(I) resistance protein CopM | 2.80 | 0.00 | -3.43 | 0.00 | -0.63 | 1.00 |
| v1_pm2247 | conserved protein of unknown function | 1.71 | 0.12 | -1.98 | 0.01 | -0.27 | 1.00 |
| v1_pm2257 | Outer membrane porin protein 32 | 1.77 | 0.01 | -2.08 | 0.00 | -0.31 | 1.00 |
| v1_pm2260 | Flagellar basal body rod protein FlgB | 4.58 | 0.00 | -4.64 | 0.00 | -0.05 | 1.00 |
| v1_pm2261 | Cadmium-transporting ATPase | 2.68 | 0.00 | -2.58 | 0.00 | 0.10 | 1.00 |
| v1_pm2263 | putative enzyme | 2.02 | 0.02 | -1.63 | 0.07 | 0.39 | 1.00 |
| v1_pm2264 | Glycosyl transferase | 2.02 | 0.00 | -2.40 | 0.00 | -0.38 | 1.00 |
| v1_pm2265 | Insertion element protein | 3.31 | 0.00 | -3.75 | 0.00 | -0.44 | 1.00 |
| v1_pm2268 | conserved protein of unknown function | 2.06 | 0.01 | -2.62 | 0.00 | -0.56 | 1.00 |
| v1_pm2281 | Cation efflux system protein CusF | 1.52 | 0.44 | -2.06 | 0.01 | -0.55 | 1.00 |
| v1_pm2286 | conserved protein of unknown function | 1.16 | 1.00 | -1.67 | 0.05 | -0.51 | 1.00 |
| v1_pm2340 | Porin | 5.83 | 0.00 | -5.80 | 0.00 | 0.03 | 1.00 |
| v1_pm2341 | protein of unknown function | 2.52 | 0.00 | -2.78 | 0.00 | -0.26 | 1.00 |
| v1_pm2343 | putative integrase/recombise | 3.71 | 0.00 | -3.67 | 0.00 | 0.04 | 1.00 |
| v1_pm2345 | conserved exported protein of unknown function | 6.86 | 0.00 | -7.02 | 0.00 | -0.16 | 1.00 |
| v1_pm2346 | CopE involved in Cu(II) resistance | 2.63 | 0.00 | -2.53 | 0.00 | 0.10 | 1.00 |
| v1_pm2348 | CopH involved in Cu(II)/Cu(I) resistance (CzcE-like protein) up with Mn | 6.81 | 0.00 | -6.53 | 0.00 | 0.28 | 1.00 |
| v1_pm2349 | Cu(II)/Cu(I) resistance protein | 6.89 | 0.00 | -11.62 | 0.00 | -4.73 | 0.01 |
| v1_pm2350 | Type II restriction endonuclease | 0.77 | 1.00 | -5.93 | 0.00 | -5.16 | 0.00 |
| v1_pm2351 | conserved protein of unknown function | 3.05 | 0.00 | -11.49 | 0.00 | -8.43 | 0.00 |
| v1_pm2352 | Copper-transporting P-type ATPase | 2.16 | 0.00 | -11.94 | 0.00 | -9.77 | 0.00 |
| v1_pm2353 | CopG protein | 2.63 | 0.00 | -11.36 | 0.00 | -8.73 | 0.00 |
| v1_pm2354 | CytoCHR1e c | 1.82 | 0.01 | -10.75 | 0.00 | -8.94 | 0.00 |
| v1_pm2355 | Copper-binding protein | 1.55 | 0.15 | -10.56 | 0.00 | -9.01 | 0.00 |
| v1_pm2356 | Copper resistance protein D | 2.53 | 0.00 | -4.51 | 0.00 | -1.97 | 1.00 |
| v1_pm2357 | Copper resistance protein C | 3.55 | 0.00 | -3.78 | 0.00 | -0.23 | 1.00 |
| v1_pm2358 | CopB outer membrane protein involved in Cu(II)/Cu(I) resistance | 2.19 | 0.01 | -2.37 | 0.00 | -0.17 | 1.00 |
| v1_pm2363 | Copper resistance protein K | 5.12 | 0.00 | -6.14 | 0.00 | -1.02 | 1.00 |
| v1_pm2364 | Cu(II)/Cu(I) resistance protein CopM | 4.08 | 0.00 | -4.63 | 0.00 | -0.56 | 1.00 |
| v1_pm2365 | conserved protein of unknown function | 2.71 | 0.00 | -2.93 | 0.00 | -0.21 | 1.00 |
| v1_pm2367 | Copper resistance protein CopV | 2.39 | 0.01 | -2.32 | 0.00 | 0.07 | 1.00 |
| v1_pm2383 | conserved protein of unknown function | -2.75 | 0.02 | 2.87 | 0.01 | 0.12 | 1.00 |
| v1_pm2526 | Outer membrane porin protein 32 | 5.34 | 0.00 | -4.82 | 0.00 | 0.52 | 1.00 |
| v1_pm2575 | protein of unknown function | -2.04 | 0.12 | 2.60 | 0.01 | 0.56 | 1.00 |
| v1_pm2601 | Coenzyme A disulfide reductase | -2.69 | 0.19 | 3.58 | 0.01 | 0.89 | 1.00 |
| v1_pm2656 | putative stress response protein | 3.00 | 0.02 | -1.74 | 0.63 | 1.26 | 1.00 |
| v1_pm2666 | conserved protein of unknown function | -3.07 | 0.00 | 3.37 | 0.00 | 0.30 | 1.00 |
| v1_pm2667 | conserved exported protein of unknown function | -2.34 | 0.00 | 2.52 | 0.00 | 0.18 | 1.00 |
| v2_0089 | sulfate adenylyltransferase, subunit 1 | -1.95 | 0.06 | 2.21 | 0.01 | 0.26 | 1.00 |
| v2_0090 | sulfate adenylyltransferase, subunit 2 | -2.37 | 0.00 | 2.67 | 0.00 | 0.29 | 1.00 |
| v2_0091 | Thioredoxin-dependent 5'-adenylylsulfate reductase | -2.76 | 0.00 | 2.85 | 0.00 | 0.09 | 1.00 |
| v2_0092 | conserved protein of unknown function | -2.74 | 0.03 | 3.15 | 0.00 | 0.41 | 1.00 |
| v2_0093 | Sulfite reductase | -2.57 | 0.00 | 2.81 | 0.00 | 0.23 | 1.00 |
| v2_0116 | ATP-binding component of ABC superfamily | -2.29 | 0.03 | 2.55 | 0.01 | 0.25 | 1.00 |
| v2_0117 | ATP-binding component of ABC superfamily | -2.00 | 0.02 | 1.99 | 0.02 | -0.01 | 1.00 |
| v2_0118 | membrane component of ABC superfamily | -2.00 | 0.04 | 1.91 | 0.06 | -0.09 | 1.00 |
| v2_0119 | membrane component of ABC superfamily | -2.24 | 0.01 | 2.22 | 0.01 | -0.02 | 1.00 |
| v2_0120 | Branched-chain amino acid ABC transporter substrate-binding protein | -2.51 | 0.01 | 2.45 | 0.01 | -0.07 | 1.00 |
| v2_0220 | pyridine nucleotide transhydrogese, beta subunit | -1.80 | 0.01 | 1.69 | 0.03 | -0.11 | 1.00 |
| v2_0379 | protein of unknown function | -2.17 | 0.42 | 2.91 | 0.03 | 0.73 | 1.00 |
| v2_0499 | Type IV pilus biogenesis and competence protein PilQ | -1.69 | 0.01 | 1.25 | 0.66 | -0.44 | 1.00 |
| v2_0500 | Pilus assembly protein PilP | -1.68 | 0.02 | 0.79 | 1.00 | -0.89 | 1.00 |
| v2_0502 | Tfp pilus assembly protein PilN | -1.81 | 0.02 | 1.21 | 1.00 | -0.60 | 1.00 |
| v2_0757 | Branched-chain amino acid ABC transporter permease | -1.78 | 0.03 | 1.52 | 0.18 | -0.26 | 1.00 |
| v2_0758 | ABC-type transporter, periplasmic component | -1.96 | 0.04 | 1.69 | 0.17 | -0.27 | 1.00 |
| v2_0792 | protein of unknown function | -1.50 | 1.00 | 2.94 | 0.03 | 1.44 | 1.00 |
| v2_0824 | conserved exported protein of unknown function | -2.02 | 0.00 | 2.10 | 0.00 | 0.09 | 1.00 |
| v2_0998 | dethiobiotin synthetase | 2.18 | 0.01 | -2.20 | 0.00 | -0.02 | 1.00 |
| v2_1210 | putative integrase | -1.64 | 0.02 | 1.48 | 0.09 | -0.16 | 1.00 |
| v2_1251 | Xanthine dehydrogese family protein subunit M | -2.49 | 0.01 | 2.57 | 0.00 | 0.07 | 1.00 |
| v2_1252 | Carbon monoxide dehydrogese large chain | -2.05 | 0.07 | 2.50 | 0.01 | 0.44 | 1.00 |
| v2_1311 | conserved exported protein of unknown function | -3.56 | 0.00 | 3.86 | 0.00 | 0.30 | 1.00 |
| v2_1312 | conserved exported protein of unknown function | -3.86 | 0.00 | 3.75 | 0.00 | -0.11 | 1.00 |
| v2_1336 | 1-Acylglycerol-3-phosphate O-acyltransferase | -2.12 | 0.00 | 1.93 | 0.00 | -0.20 | 1.00 |
| v2_1354 | Permease of the Major Facilitator Superfamily | -2.66 | 0.01 | 2.76 | 0.00 | 0.10 | 1.00 |
| v2_1463 | 2',3'-cyclic-nucleotide 2'-phosphodiesterase/3'-nucleotidase | -2.46 | 0.00 | 2.59 | 0.00 | 0.13 | 1.00 |
| v2_1464 | Sel1 repeat family protein | -2.33 | 0.01 | 2.57 | 0.00 | 0.25 | 1.00 |
| v2_1569 | Type IV pilus transmembrane protein PilJ | -1.72 | 0.03 | 1.57 | 0.08 | -0.15 | 1.00 |
| v2_1571 | putative metabolite transport protein NicT | -1.58 | 0.06 | 1.68 | 0.02 | 0.10 | 1.00 |
| v2_1602 | Zonular occludens toxin | -1.14 | 1.00 | 1.69 | 0.05 | 0.55 | 1.00 |
| v2_1609 | protein of unknown function | -2.10 | 0.08 | 2.52 | 0.01 | 0.42 | 1.00 |
| v2_1658 | conserved protein of unknown function | 1.18 | 1.00 | -1.76 | 0.04 | -0.59 | 1.00 |
| v2_1660 | UDP-glucose 6-dehydrogese | 1.45 | 0.23 | -1.68 | 0.03 | -0.24 | 1.00 |
| v2_1813 | conserved protein of unknown function | -1.69 | 0.08 | 2.01 | 0.01 | 0.32 | 1.00 |
| v2_1819 | conserved protein of unknown function | -2.82 | 0.00 | 3.01 | 0.00 | 0.18 | 1.00 |
| v2_2018 | transposase | 1.67 | 0.04 | -2.02 | 0.00 | -0.34 | 1.00 |
| v2_2063 | putative malate transporter YflS | -1.72 | 0.01 | 1.82 | 0.00 | 0.11 | 1.00 |
| v2_2154 | D-specific glutamate dehydrogese | -1.82 | 0.04 | 2.12 | 0.00 | 0.30 | 1.00 |
| v2_2155 | putative transcriptiol regulator, MarR family | -1.84 | 0.01 | 1.66 | 0.05 | -0.18 | 1.00 |
| v2_2156 | protein of unknown function | -2.04 | 0.22 | 2.75 | 0.01 | 0.71 | 1.00 |
| v2_2202 | ABC transporter permease | -2.22 | 0.01 | 2.57 | 0.00 | 0.35 | 1.00 |
| v2_2203 | Branched-chain amino acid ABC-type transport system | -1.59 | 0.03 | 1.63 | 0.02 | 0.04 | 1.00 |
| v2_2206 | ATP-binding component of ABC superfamily | -1.91 | 0.05 | 2.29 | 0.00 | 0.38 | 1.00 |
| v2_2232 | periplasmic-binding component of ABC superfamily | -1.93 | 0.02 | 1.71 | 0.07 | -0.22 | 1.00 |
| v2_2239 | sulfate transport protein (ABC superfamily, membrane) | -1.72 | 0.17 | 2.04 | 0.02 | 0.32 | 1.00 |
| v2_2378 | conserved protein of unknown function | -1.33 | 0.83 | 1.71 | 0.03 | 0.39 | 1.00 |
| v2_2388 | Esterase | -1.92 | 0.41 | 2.74 | 0.01 | 0.82 | 1.00 |
| v2_2417 | conserved protein of unknown function | -1.51 | 0.29 | 1.76 | 0.04 | 0.25 | 1.00 |
| v2_2423 | MarR family transcriptiol regulator | -2.67 | 0.00 | 2.68 | 0.00 | 0.01 | 1.00 |
| v2_2447 | Sensor histidine kise | -2.90 | 0.03 | 3.42 | 0.00 | 0.52 | 1.00 |
| v2_2448 | Transcriptiol regulatory protein tctD | -8.77 | 0.00 | 8.29 | 0.00 | -0.48 | 1.00 |
| v2_2522 | conserved exported protein of unknown function | -2.01 | 0.07 | 2.61 | 0.00 | 0.60 | 1.00 |
| v2_2525 | putative Histidine kise | -2.11 | 0.01 | 2.28 | 0.00 | 0.17 | 1.00 |
| v2_2526 | Transcriptiol regulatory protein FixJ | -2.10 | 0.01 | 2.22 | 0.00 | 0.12 | 1.00 |
| v2_2527 | protein of unknown function | -2.71 | 0.10 | 2.97 | 0.03 | 0.26 | 1.00 |
| v2_2528 | Response regulator, LuxR-family | -2.71 | 0.11 | 3.24 | 0.02 | 0.53 | 1.00 |
| v2_2530 | Cation/multidrug efflux system, mebrane-fusion component | -1.42 | 0.75 | 1.88 | 0.03 | 0.45 | 1.00 |
| v2_2531 | Efflux pump membrane transporter BepE | -1.65 | 0.12 | 1.96 | 0.01 | 0.31 | 1.00 |
| v2_2533 | conserved exported protein of unknown function | -1.94 | 0.08 | 2.18 | 0.01 | 0.24 | 1.00 |
| v2_2534 | conserved exported protein of unknown function | -1.51 | 0.42 | 1.98 | 0.02 | 0.47 | 1.00 |
| v2_2595 | response regulator in two-component regulatory system | 5.81 | 0.00 | -9.18 | 0.00 | -3.37 | 0.00 |
| v2_2596 | Sensor protein | 5.62 | 0.00 | -14.14 | 0.00 | -8.52 | 0.00 |
| v2_2612 | Glutamate decarboxylase | -1.34 | 1.00 | 2.14 | 0.02 | 0.80 | 1.00 |
| v2_2634 | conserved protein of unknown function | -2.85 | 0.02 | 2.78 | 0.03 | -0.07 | 1.00 |
| v2_2711 | ABC-type transporter, periplasmic component: HAAT family | -2.36 | 0.01 | 2.40 | 0.01 | 0.04 | 1.00 |
| v2_2725 | TRAP-type transporter, periplasmic component / DctP alogue | -1.87 | 0.01 | 2.11 | 0.00 | 0.24 | 1.00 |
| v2_2767 | conserved protein of unknown function | -2.07 | 0.01 | 2.18 | 0.00 | 0.11 | 1.00 |
| v2_2787 | conserved protein of unknown function | -1.76 | 0.77 | 2.62 | 0.03 | 0.85 | 1.00 |
| v2_2823 | protein of unknown function | -2.30 | 0.24 | 3.18 | 0.01 | 0.88 | 1.00 |
| v2_2824 | FAA1, Long-chain acyl-CoA synthetase (fragment) | -2.19 | 0.62 | 3.15 | 0.03 | 0.96 | 1.00 |
| v2_2908 | Bifunctiol aspartate aminotransferase and L-aspartate beta-decarboxylase | -2.26 | 0.02 | 2.55 | 0.01 | 0.28 | 1.00 |
| v2_2957 | protein of unknown function | -1.72 | 1.00 | 2.68 | 0.03 | 0.96 | 1.00 |
| v2_3058 | conserved protein of unknown function | -2.10 | 0.38 | 2.72 | 0.04 | 0.62 | 1.00 |
| v2_3302 | Benzoate--CoA ligase | -1.24 | 1.00 | 2.27 | 0.02 | 1.03 | 1.00 |
| v2_3487 | regulator protein that represses frmRAB operon | 1.60 | 0.48 | -2.25 | 0.01 | -0.65 | 1.00 |
| v2_3753 | putative antioxidant protein | -1.98 | 0.01 | 1.89 | 0.01 | -0.09 | 1.00 |
| ^a^NA4 (CmetNA4_) locus tag based on MaGe annotation (http://www.genoscope.cns.fr/agc/microscope/mage/index.php); ^b^Log2 fold change in non-selective growth conditions (n = 3). | | | | | | | |

| Supplementary Table 4: Inventory of two-component regulatory systems in *C. metallidurans* NA4. | | | | | | | |
| --- | --- | --- | --- | --- | --- | --- | --- |
|  | Replicon^a^ | | | | | | Total |
|  | CHR1 | CHR2 | pA | pB | pA | pD |  |
| **Histidine kinase** | **30** | **32** | **1** |  |  | **1** | **64** |
| CheA | 1 | 2 |  |  |  |  | 3 |
| Classic | 26 | 23 | 1 |  |  | 1 | 51 |
| Hybrid | 3 | 4 |  |  |  |  | 7 |
| Unorthodox |  | 3 |  |  |  |  | 3 |
| **Response regulator** | **40** | **43** | **2** | **1** |  | **1** | **87** |
| AmiR_NasR | 1 |  |  |  |  |  | 1 |
| CheB |  | 2 |  |  |  |  | 2 |
| CheV |  | 1 |  |  |  |  | 1 |
| CheY | 7 | 6 | 1 |  |  |  | 14 |
| LytTR | 1 | 1 |  |  |  |  | 2 |
| NarL | 9 | 14 |  |  |  | 1 | 24 |
| NtrC | 4 |  |  |  |  |  | 4 |
| OmpR | 14 | 17 | 1 | 1 |  |  | 33 |
| PleD |  | 1 |  |  |  |  | 1 |
| PrrA | 2 |  |  |  |  |  | 2 |
| unclassified | 2 |  |  |  |  |  | 2 |
| VieA |  | 1 |  |  |  |  | 1 |
| ^a^CHR1: chromosome; CHR2: chromid; pA: pNA4_A; pB: pNA4_B; pC: pNA4_C; pD: pNA4_D | | | | | | | |

## Supplementary Figures


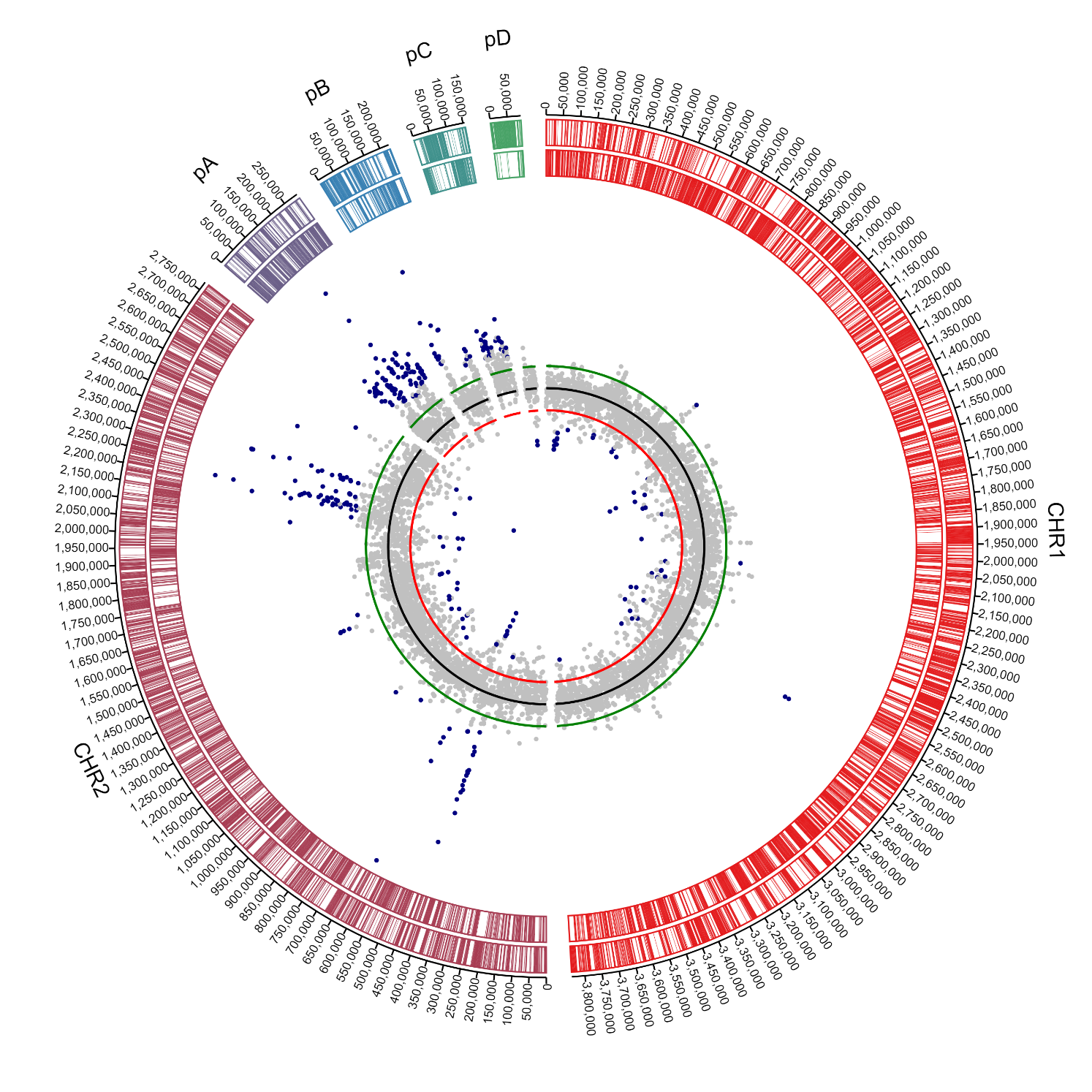


**Supplementary Figure 1.** Scatter plot of RNA-seq-derived gene expression of *C. metallidurans* NA4S compared to its parental strain in non-selective conditions. Dots (blue p<0.05) represent log2 ratios with red, black and green lines corresponding to -1, 0 and 1, respectively.


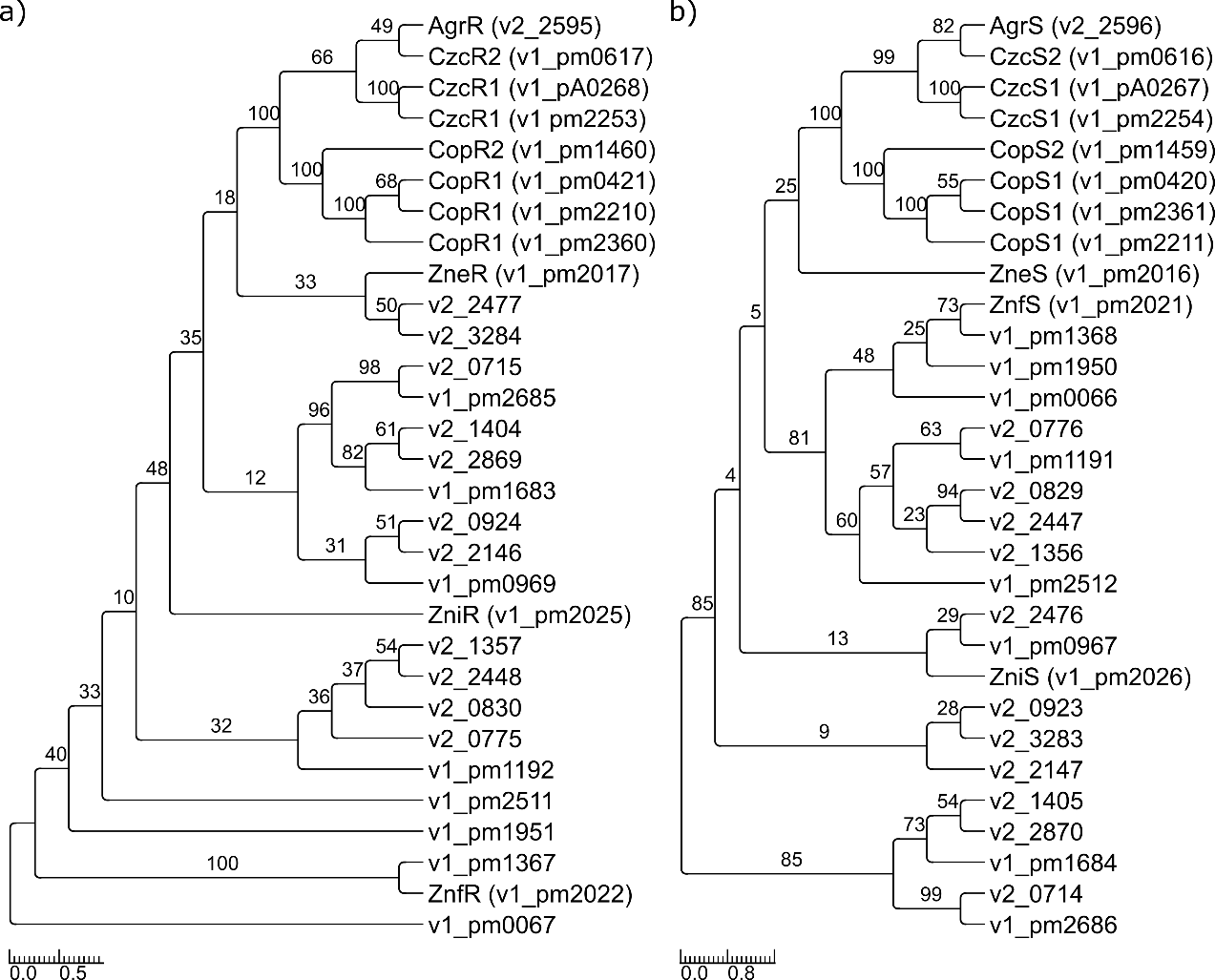


**Supplementary Figure 2.** Neighbor Joining phylogenetic tree (Jukes-Cantor model) of RRs (a) and HKs (b) of complete TCSs with OmpR-family RRs in *C. metallidurans* NA4. The numbers above branches indicate bootstrap support values (1000 replicates).


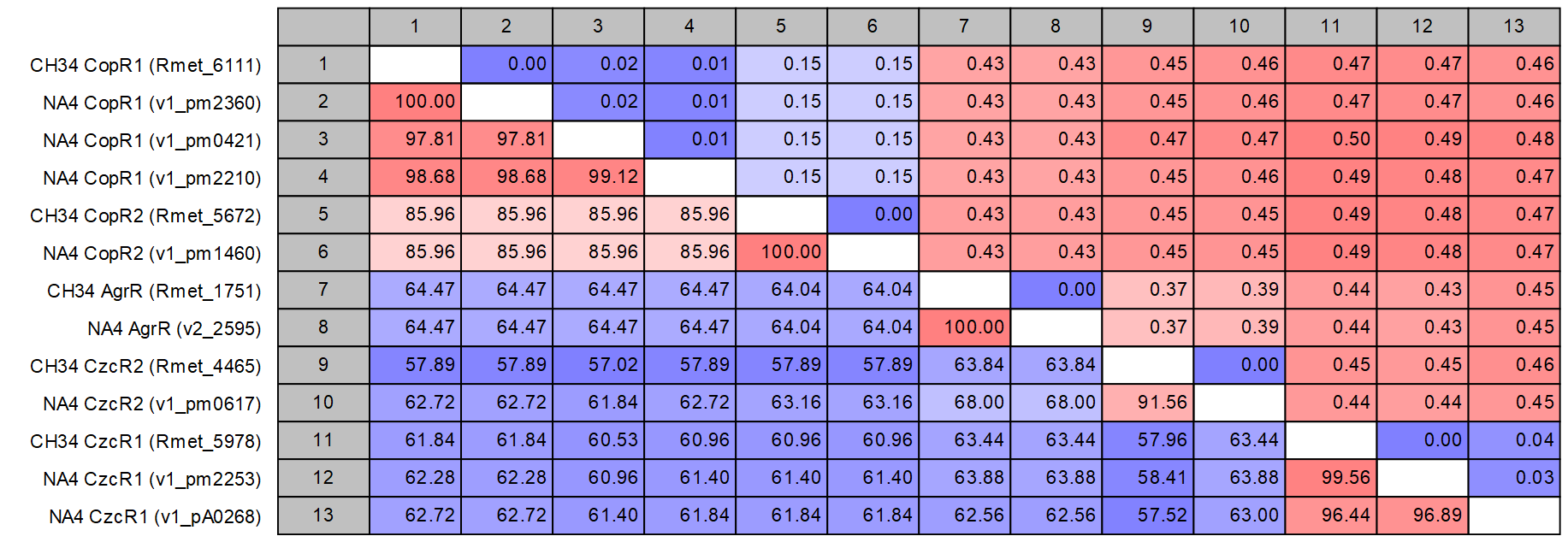


**Supplementary Figure 3.** Pairwise comparison (upper: distance; lower: percent identity) of selected response regulators from *C. metallidurans* NA4 and CH34.


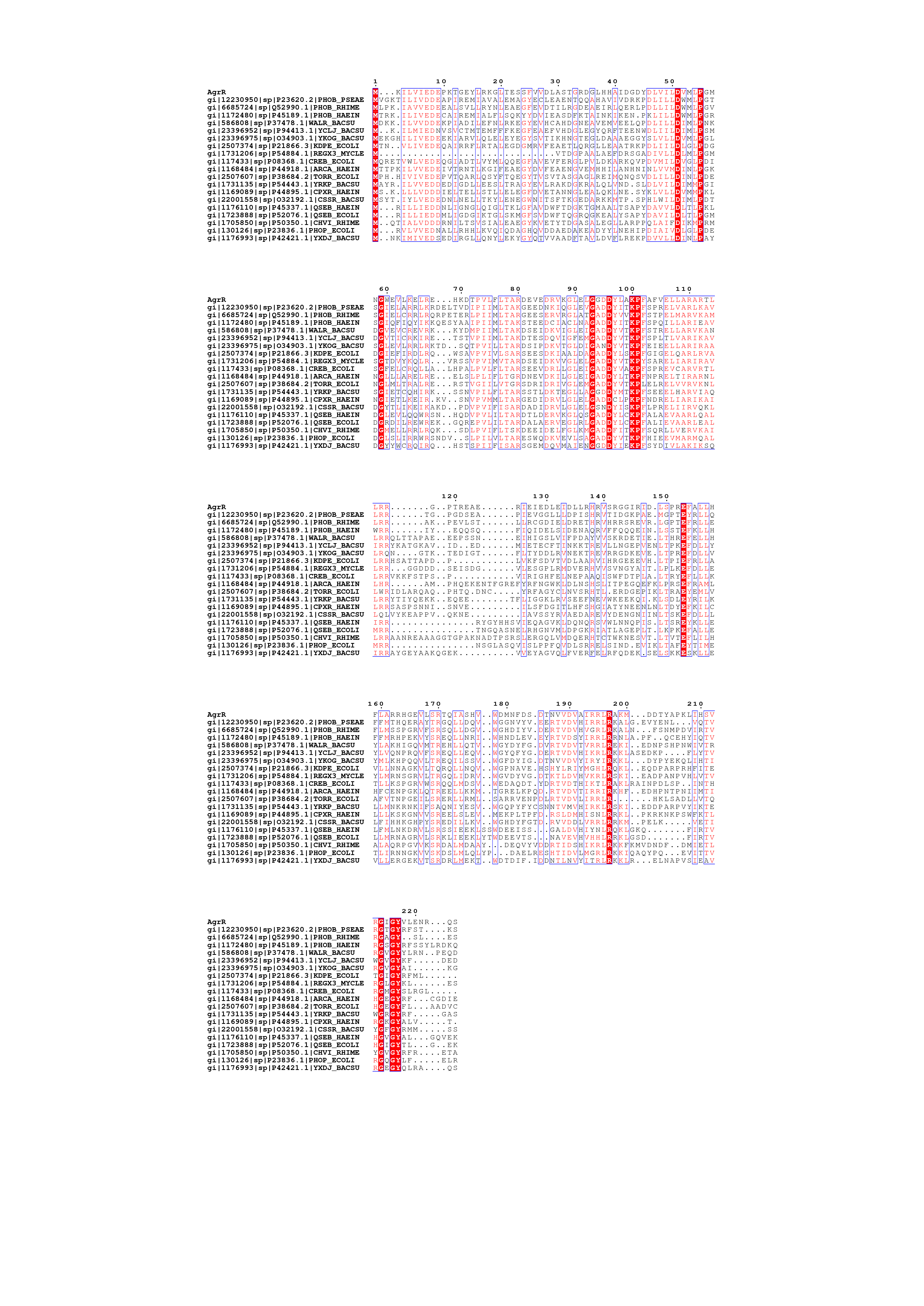


**Supplementary Figure 4.** Alignment of AgrR with representatives from the OmpR-family.


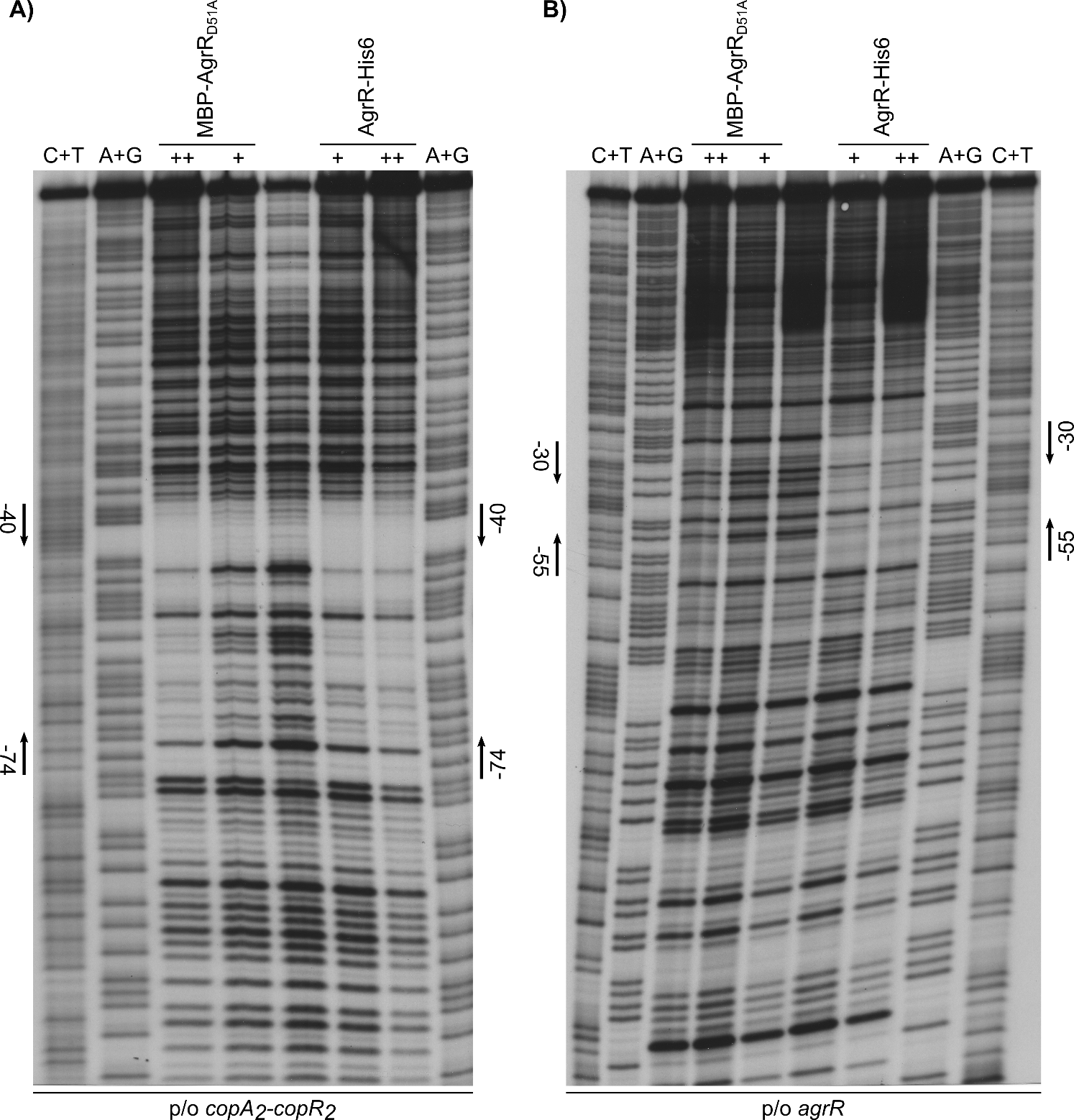


**Supplementary Figure 5.** DNase I footprinting with MBP-AgrR_D51A_ and AgrR-His6 binding to the intergenic *pcoA_2_-copR_2_* region (coding strand for *copA_2_* revealed) (**A**) and the *agrR* control region (coding strand revealed) (**B**). A+G and C+T are the chemical sequencing ladders. Protein concentrations used are 2.9 (+) and 7.25 µM (++) for MBP-AgrR_D51A_ and 20 (+) and 40 µM (++) for AgrR-His6. Black colored arrows delimit the regions of protection.
